# Unveiling Human Non-Random Genome Editing Mechanisms Activated in Response to Chronic Environmental Changes: I. Where Might These Mechanisms Come from and What Might They Have Led To?

**DOI:** 10.3390/cells9112362

**Published:** 2020-10-27

**Authors:** Loris Zamai

**Affiliations:** 1 Department of Biomolecular Sciences, University of Urbino Carlo Bo, 61029 Urbino, Italy; loris.zamai@uniurb.it; Tel./Fax: +39-0722-304-319; 2 National Institute for Nuclear Physics (INFN)-Gran Sasso National Laboratory (LNGS), 67100 Assergi, L’Aquila, Italy

**Keywords:** virus, pollutant, environmental stress, retrotransposons, mutagenic enzymes, symbiosis, biological plasticity, altruistic biological behaviour, transgenerational non-Mendelian gene transmission, fractal systems

## Abstract

This article challenges the notion of the randomness of mutations in eukaryotic cells by unveiling stress-induced human non-random genome editing mechanisms. To account for the existence of such mechanisms, I have developed molecular concepts of the cell environment and cell environmental stressors and, making use of a large quantity of published data, hypothesised the origin of some crucial biological leaps along the evolutionary path of life on Earth under the pressure of natural selection, in particular, (1) virus–cell mating as a primordial form of sexual recombination and symbiosis; (2) Lamarckian CRISPR-Cas systems; (3) eukaryotic gene development; (4) antiviral activity of retrotransposon-guided mutagenic enzymes; and finally, (5) the exaptation of antiviral mutagenic mechanisms to stress-induced genome editing mechanisms directed at “hyper-transcribed” endogenous genes. Genes transcribed at their maximum rate (hyper-transcribed), yet still unable to meet new chronic environmental demands generated by “pollution”, are inadequate and generate more and more intronic retrotransposon transcripts. In this scenario, RNA-guided mutagenic enzymes (e.g., Apolipoprotein B mRNA editing catalytic polypeptide-like enzymes, APOBECs), which have been shown to bind to retrotransposon RNA-repetitive sequences, would be surgically targeted by intronic retrotransposons on opened chromatin regions of the same “hyper-transcribed” genes. RNA-guided mutagenic enzymes may therefore “Lamarkianly” generate single nucleotide polymorphisms (SNP) and gene copy number variations (CNV), as well as transposon transposition and chromosomal translocations in the restricted areas of hyper-functional and inadequate genes, leaving intact the rest of the genome. CNV and SNP of hyper-transcribed genes may allow cells to surgically explore a new fitness scenario, which increases their adaptability to stressful environmental conditions. Like the mechanisms of immunoglobulin somatic hypermutation, non-random genome editing mechanisms may generate several cell mutants, and those codifying for the most environmentally adequate proteins would have a survival advantage and would therefore be Darwinianly selected. Non-random genome editing mechanisms represent tools of evolvability leading to organismal adaptation including transgenerational non-Mendelian gene transmission or to death of environmentally inadequate genomes. They are a link between environmental changes and biological novelty and plasticity, finally providing a molecular basis to reconcile gene-centred and “ecological” views of evolution.

## 1. Waiting for Beneficial Random Mutations within the Vast Eukaryotic Cell Genome: Is It Like “Waiting for Godot”?

### 1.1. Random Mutations: A Weak Point in the Modern Synthesis of Evolution

The modern synthesis of evolution is a gene-centred theory based on Darwinian natural selection and Mendelian genetics. Darwinian selection is a good mechanism for excluding inadequate genomes/organisms, and Mendelian genetics ensures allele inheritance from generation to generation, while limiting the appearance of new characteristics. These mechanisms are not effective in generating biological variability and novelties, which are believed to depend on a relatively constant rate of random mutations. However, random mutations (e.g., during DNA replication) may generate not only advantageous or neutral effects, but also detrimental ones, ultimately yielding many aborted alternatives that can only be managed by large highly proliferating populations such as viruses and bacteria. Viruses and bacteria can take advantage of random mutations not only because of their rapid proliferation, but also because of their low DNA content, which limits the number of random detrimental mutations per biological entity. Indeed, there is a strong inverse correlation between the mutation rate during DNA replication and genome size across all replication systems, suggesting that any increase in DNA content and complexity requires a reduction in potentially deleterious mutations [1,2]. Hence, the lowest error rates per nucleotide are reported for eukaryotes, while the highest mutation rates are observed in viroids, which are short single-stranded circular RNA without a protein coat and likely the first virus-like structures and survivors of the hypothetical “RNA world” stage in the evolutionary history of life on Earth [2,3]. This evidence suggests that error rates have been progressively reduced over evolutionary time and biological complexity [1,2], raising the possibility that new evolutionary mechanisms, able to generate novelties, have been developed during the evolution of increasingly complex organisms. Indeed, bacteria, which have a larger genome (10 to 100 times larger) than viruses [1,2], have developed stress induced mutagenesis (SIM) mechanisms [4,5], which are induced by environmental stresses and represent a quasi-Lamarckian phenomenon [6]. The SIM is mainly mediated by error-prone DNA duplicating and/or repair systems, which allow bacteria to accelerate mutation rates and therefore to enhance their ability to adapt to environmental challenges [4,5]. Indeed, SIM has been hypothesised to be an adaptive strategy [7]. However, along the vast genome of relatively “slow-proliferating” eukaryotic cells, it is very unlikely that random mutations occurring during DNA duplication and/or repair could produce mutations that are advantageous (or neutral) to adaptation without also producing detrimental ones, and therefore a myriad of unproductive eukaryotic attempts. This is likely the reason why they were selected with the lowest mutation rate per genome replication. It is therefore difficult to explain how only random mutations could have led to the sophisticated molecular entanglements of eukaryotic cells and to the huge and expanding number of species found on Earth today. Indeed, random mutations represent a weak point in the modern synthesis of evolution, and mathematicians have predicted the existence of non-random mutation mechanisms [8] and non-random genome manipulation mechanisms have also been proposed [9,10,11,12].

### 1.2. The Complex Protein–Protein Steric Interactions of Eukaryotic Cells Originate from the Translation of Unrelated Genes: The Needs of Gene Editing Feedback Mechanisms

The complexity of the molecular signals that regulate the life of eukaryotic cells is well known. Interactions among proteins, from the cell surface to the cell nucleus and back, function on the basis of complex steric interactions regulated by weak inter- and intra-molecular bonds. Variable affinities among different inter- and intra-molecular domains generate a complex web of allosteric communications that makes the extraordinary “phenomenon” of eukaryotic cell life possible. Interestingly, the interaction affinities of cellular and extracellular molecules vary according to context. For example, immunoglobulins usually bind to pathogens at high affinities, while TcR-MHC binding works at intermediate affinities, and sometimes even a loss of recognition can be advantageous, as in the case of CCR5-Delta32 mutation, which provides resistance to human immunodeficiency virus type I (HIV-1) [13]. How can these recognition affinities be adjusted to the needs of the cell and organism?

Importantly, unlike poly-cistronic prokaryotic genes, most of the sophisticated entanglements among eukaryotic cell molecules originate from the translation of unrelated genes, which do not “know” that their translational products will sterically interact with each other in such a complex and functional fashion! This aspect cannot be accounted for by the induction of indiscriminate random mutations along the vast eukaryotic DNA molecule, pointing to the existence of gene editing feedback mechanisms able to surgically generate mutations on the genes of inefficient proteins, leaving the rest of the genome intact. The death of cells bearing inadequate protein versions and the survival of cells bearing the fittest mutation(s) able to increase protein efficiency might then determine the “selection” of the inheritable gene version(s). Indeed, a non-random mutation mechanism with similar activity has already been described in adaptive immunity during somatic hypermutation (SHM) of immunoglobulins (Igs) [9,10]. However, it would appear that known mechanisms of adaptation are unable to explain the above-mentioned observations regarding other eukaryotic genes.

### 1.3. Environmental Changes Drive Organisms to Evolve Rapidly: The Evolvability Trait Was Likely Selected Early on in the Evolution of Life

In contrast to lifeless planets that have relatively stable environmental conditions, over the last four billion years, the biological components present on Earth have “stimulated” each other, producing enormous and progressive environmental changes (environmental plasticity), which are in addition to the changes brought about suddenly by meteorite impacts, earthquakes and volcanic eruptions [14,15]. Indeed, soon after the expansion of life on Earth, biological structures started to produce metabolic waste products and in geographical regions with physicochemical barriers limiting waste dispersion, they were likely poisoned by their own catabolites (bio-pollutants). This event could drive biological structures to develop evolvability mechanisms that made it possible to plastically and rapidly adapt to and manage increasing concentrations of environmental pollutants (new molecules able to sterically interfere with cellular structures) of biological origin. In Earth’s changeable environment (due either to increasing biological catabolites or to suddenly appearing abiotic compounds), the evolvability trait was likely a crucial characteristic for the survival of life, and it was probably selected early on as life expanded. Thus, it is likely that environmental (bio)pollutants accumulated in specific geographical regions pushed biological species to plastically change into new species (through selected evolvability mechanisms), which, in turn, produced new biological pollutants that gradually generated new niches, and hence, new adaptations, in a continuous evolutionary process. In this “never-ending history”, some biotic (i.e., O_2_ was a plant pollutant), but also abiotic pollutants became fundamental bricks for subsequent biological structures, and abiotic components progressively became biotic ones.

### 1.4. Why Should Eukaryotic Organisms Survive Viruses?

Viruses as well as pollutants cause (environmental) pressure for eukaryotic organisms to adapt. However, unlike viruses and bacteria, eukaryotic cells in organisms possess a large DNA sequence and transmit their genes to their progeny relatively slowly. During evolution, rapidly evolving viruses have repeatedly threatened the survival of seemingly slowly evolving eukaryotic organisms. Despite these continuous viral challenges, organisms have not only been able to survive but also to expand until the present day.

It is well known that during viral infection, viruses can inject their genetic material into host cell genomes. Viral gene translation then provides instructions for their rapid replication and the building of their components. Once mature viruses are formed, they break open the cell and move on to infect other cells. Moreover, error-prone virus replication rapidly generates a repertoire of viral variants that enhances the ability of viruses to adapt and elude cell and organism immune controls. So how is it possible that eukaryotic organisms did not succumb to these rapidly adapting microorganisms and actually managed to expand explosively? Have they developed potent mechanisms able to counter the rapid evolution of viruses “in real time”?

It is likely that complex eukaryotic organisms developed after viruses and prokaryotes, and therefore, in their presence. This means that eukaryotic cells were selected to be able to co-evolve with and manage rapidly adapting and proliferating microorganisms. Indeed, mitochondria and chloroplasts are examples of microorganisms which have not only been managed but also exploited by eukaryotic cells [11,16]. In this regard, Eco-Evo-Devo theory teaches us that symbiosis between eukaryotic organisms and their persistent microorganisms seems to be a rule rather than an exception [17].

### 1.5. The Open Questions of the “Ecological” and Punctuated Equilibrium Views of Evolution

The need to incorporate ecology and development into modern evolutionary theory is highlighted by ecological evolutionary developmental biology (Eco-Evo-Devo [17]), the extended evolutionary synthesis [18] and the Read-Write genome evolution [11]. Indeed, these theories show how environmental changes contribute to shape the production of a range of phenotypes (called developmental plasticity) promoting biological adaptation and only subsequently to environmentally induced phenotypes, they are selected by environmental conditions and ultimately “accommodated” into the genome, becoming stably inheritable traits (a process called genetic accommodation). However, the dynamic interplay between environment and biological entities yields not only developmental plasticity (environmentally induced phenotypes) and genetic accomodation, but also developmental symbiosis between organisms and microorganisms, leading to the generation of “microbial organs” and “holobionts” [11,17]. On the other hand, developing phenotypes affect their niches (niche construction) driving additional biological variation. Notably, when developmental plasticity generates new metabolic pathways, new biological waste products tend to be progressively accumulated and geographical regions with physicochemical barriers that keep their own waste products (by limiting their diffusion outside the borders) might lead to the construction of new ecological niches. Such waste products may not be toxic for a long time until their increasing concentrations begin to interfere with biological structures/activities, becoming toxic and “stressing” the ecological niche. However, toxic concentrations of waste products require a rapid biological adaptation, which can occur through the generation of species able to manage the “new” waste products. Therefore, cyclic phases of rapid speciation (expression of developmental plasticity) followed by longer periods of species stability are expected differently depending on the geographical region. Indeed, based on fossil records, the theory of punctuated equilibrium proposes that biological evolution is not constant, but rather, is characterised by rapid accelerations with speciations (clades) followed by long periods of stasis [19]. In line with this theory, two recent well-known events come to mind: the chromosomal speciation in mice observed in Seveso (Italy) in the wake of a major industrial accident involving dioxin contamination [20] and the rapid development of the black form of the British peppered moths in areas with heavy industrial pollution [21]. It is important to note that the rapid biological evolution that occurred in these cases was associated with previous deep environmental changes, suggesting a cause-effect relationship. However, it is difficult to imagine that random mutations and selection could have generated such rapid organism adaptations to these deep environmental changes. Of note, under stress conditions, the mutation rate substantially increases (e.g., under SIM) and the probability of multiple adaptive mutations and saltational evolution (a sudden multimutation leap potentially leading to speciation and macroevolution) becomes more likely to occur [7]. Indeed, rapid genetic adaptations of eukaryotic organisms induced by environmental stress conditions have been widely documented and environmentally driven non-random genome variations mediated by different mechanisms (such as DNA mutagenic and/or methylating/de-methylating enzymes and/or transposable elements) have been proposed to explain some rapid biological adaptations [9,10,11,12,22,23]. However, these hypothesised mechanisms do not exhaustively describe the specific molecular links that mediate the causal relationship between environmental stress conditions and the ability to rapidly induce adaptive DNA mutations in specific genomic regions (while sparing the rest of the genome). Hence, the molecular mechanisms underlying the genetic variations of such environmentally induced traits are still largely unknown, and researchers are still working to unveil the molecular rules governing the genetic adaptations of organisms to changes in their environmental niches. This is the missing link between ecological and gene-centred views of evolution. In this regard, I hypothesise that eukaryotic cells may possess potent, yet to be discovered mechanisms of adaptation to respond to environmental challenges. Such mechanisms would be able to non-randomly choose a few specific genes in which to “Lamarckianly” generate mutations (new acquired characteristics), and subsequently, Darwinian selection would determine the survival and inheritability of only the advantageous mutations. Among the known non-random genome mechanisms, there is a mechanism restricted to the immune system that perfectly matches these characteristics: the somatic hypermutation of immunoglobulins that occurs in memory B cells [24].

### 1.6. Immunoglobulin Somatic Hypermutation as a Mechanism Model for Eukaryotic Gene/Cell Evolution

This process starts when B cells produce antibodies with a low affinity for pathogenic antigens. They are inadequate to eradicate the pathogen and its chronic presence (environmental challenge) induces chronic transcription of Ig genes and proliferation of B cells bearing low affinity antibodies. Starting from a single B cell, this mechanism, mediated by the activation-induced deaminase (AID) mutagenic enzyme, generates a range of B cell mutants bearing slightly different Ig gene versions (genetic plasticity) coding for different immunoglobulin variants (phenotypic plasticity). Among the different cell mutants, those producing antibodies with the highest affinity for new and persistent pathogenic antigens are selected [24], ultimately eradicating the pathogen from the host organism and increasing the chance of organism survival. Although a putative molecular mechanism mediated by environmental antigens was already proposed to induce non-random mutations in Ig genes by Steele and colleagues [9,10], the molecular mechanisms that underlie the link between environmental changes and non-random genome editing mechanisms that rapidly overcome environmental challenges (particularly in non-Ig genes) are still largely unknown.

Interestingly, this mechanism represents an example of “developmental plasticity”, in which a single cell genome can generate different cell phenotypes as a function of intra-organismal “environmental” stress conditions (the pathogen presence). In this case, immunoglobulin gene “plasticity”, activated in response to the chronic presence of pathogenic antigens, provides the raw material for the environmental selection of phenotypes, ultimately yielding new inheritable and “environmentally independent” versions of Ig genes, what Waddington called the genetic assimilation of acquired characters [25].

## 2. Are Viral Nucleic Acid Insertions in the Prokaryotic Cells Always Harmful or Can They Also Be Useful to Prokaryotic Cells? The Example of CRISPR-Cas Systems

### 2.1. Selection of Virus–Cell Mating Pairs: A Primordial Form of “Sexuality” and Symbiosis

**Figure 1:**
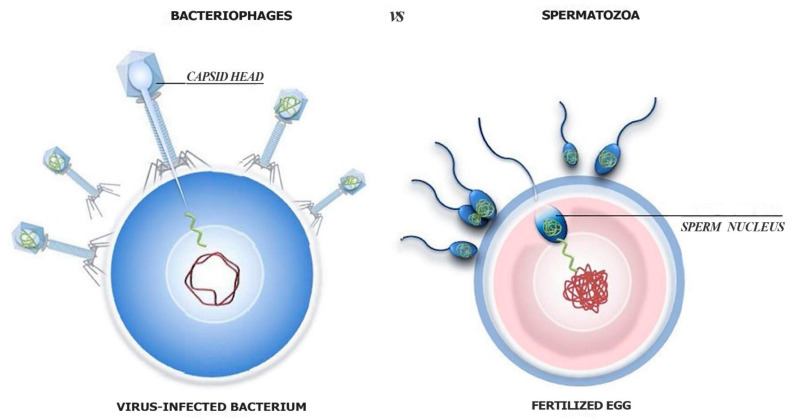
Virus–cell mating pairs: a primordial form of “sexuality”. Bacteriophages and male gametes have the ability to insert new genetic material (novelties) into prokaryotic cells and oocytes, respectively. (For reference, see the text.).

At a certain point after life appeared, among the myriad of primordial cells and viruses, it is likely that some of them started to bind to one another through surface molecule interactions, giving rise to specific viral tropism. Subsequently, some viruses acquired the ability to insert new genetic material into prokaryotic cells, similarly to how spermatozoa insert their genetic material into oocytes (see Figure 1).

Indeed, viruses usually have a preferential prokaryotic or eukaryotic tissue target, a species-specific and persistent virus interaction (tropism) with their cell host, which can represent a primordial and harmful form of mating and “sexual” recombination. In fact, an uncontrolled insertion of nucleic acid material produces cell gene disruptions, and lethally infected cells also stop spreading the virus. However, among the myriad of aborted attempts, the pairs of cells and viruses that incidentally generated viral nucleic acid insertions with low cell toxicity had a better chance of survival, which gave them a selective advantage. Moreover, a controlled horizontal insertion of new genetic material into the cell genome during viral infection could be a rich source of new genes and new nucleotide sequences for adaptive innovation of host cells. It is indeed plausible that in a changeable environment, like that on Earth, the viral provision of new biological tools enhanced cell adaptability, giving infected cells a better chance of survival than uninfected (“sterile”) cells. Although at different levels (eukaryotic vs. prokaryotic) of organisation and complexity, current specialised mechanisms of organismal sexual reproduction functionally resemble those of virus–cell mating pairs (see Figure 1). They would represent mechanisms of nucleic acid recombination leading to variability/novelty that were selected (among myriads of attempts) for their reduced “toxicity”. Of note, controlled viral insertions may not only have conferred an advantage to cells, but also allowed viruses to survive within their host-cells, representing a sort of cell-virus agreement, a symbiosis, naturally selected among countless unproductive struggles. In line with this hypothesis is the evidence that co-evolution and symbiosis of organism hosts with their microbial communities are the rule and not the exception [17]. In addition, bacterial sequences integrated through horizontal gene transfer in the genome of their (multicellular organism) hosts and able to pass to offspring have also been documented [11,16,26]. How eukaryotic organisms began to interact with a specific set of beneficial microorganisms, while avoiding dangerous microorganisms is unclear [17]; however, a selective mechanism similar to the one described above (between bacteriophages and prokaryotic cells) can be hypothesised.

### 2.2. The Meanings of Virus Fragments in Lamarckian CRISPR-Cas Systems

During the evolution of the interplay between prokaryotic cells and viruses, an important improvement in prokaryotes took place when they developed compartments specialised in storing viral nucleic acids, the CRISPR (clustered, regularly interspaced, short, palindromic repeats)-Cas (CRISPR-associated) systems. From a functional point of view, CRISPR-Cas systems constitute adaptive immune organelles segregated far from the cell genome (see Box 1).

Box 1 **Adaptive immune organelles of prokaryotic cells: CRISPR-Cas systems.**

CRISPR-Cas systems represent prokaryotic organelles specialised in storing viral nucleic acids, which have been discovered in most archaeal species and half of bacteria [27,28]. During the first bacteriophage infection, the foreign DNA is processed by cell restriction enzymes in viral fragments. Some of these fragments, called spacers, are integrated within the repeats of CRISPR arrays of prokaryotic cells. The spacer acquisition, also known as adaptation, allows the cells to store viral sequences and to retain the memory of virus contact, providing a heritable record of previous viral exposures. In the case of reinfection, the CRISPR array is transcribed into long CRISPR RNAs (pre-crRNAs) of alternating repeat–spacer sequences and then “spliced” into short CRISPR RNAs (crRNAs). The crRNAs form complexes with Cas endonucleases, which are encoded in an adjacent operon. Repeats are necessary for crRNA binding to Cas proteins, while spacers give the specificity to target Cas endonucleases against viral nucleic acids for their destruction [29]. Spacers contain a 20-nucleotide guide sequence able to bind to the re-entered foreign DNA/RNA in a complementary sequence-specific manner [29], and the target specificity is likely assured by the uniqueness of the viral sequences, which are not expressed in the host cell genome. Regarding the control of CRISPR-Cas transcription, evidence suggests that this is elicited by cell envelope stresses, typically generated during viral infection [30]. This link is functional because it allows both the integration of viral nucleic acid into CRISPR-Cas systems during the first virus invasion and the transcription of repeat-spacers and Cas endonucleases following virus re-challenge. Recently, it has also been observed that some Cas subunits form a complex with crRNA (called Cascade, CRISPR-associated complex for antiviral defence), which binds the promoter region of the Cas operon and functions as a transcriptional repressor by inhibiting Cas gene transcription through a negative feedback loop [31]. Upon viral infection, Cascade complex redistribution towards the viral genome would “sense” viral infection and relieve transcriptional repression, allowing a rapid transcriptional response to reinfection [31].

On the other hand, from a structural standpoint, the CRISPR-Cas systems consist of two (usually adjacent) components: the CRISPR locus composed of alternating repeat-spacer non-coding sequences and a poly-cistronic gene-coding for a series of Cas proteins [27,28]. It should be noted that the CRISPR locus can be both transcribed and “spliced”, but differently from Cas genes, it cannot be translated into proteins. CRISPR-Cas systems call to mind a primordial form of the “intron-exon” gene structure, typical of eukaryotic cells, in which the CRISPR locus represents the prototype of introns. The simple structure of CRISPR-Cas systems, formed by an operon and repetitive sequences, points to its viral origin. Indeed, the manner of integration of new viral fragments is reminiscent of viral integrases and transposases [29], and several findings suggest that prokaryotic Cas endonucleases were derived from the “domestication” of viral transposases [32,33]. In line with these observations, bacteriophages with their own CRISPR-Cas systems have been described [34], raising the possibility of horizontal transpositions of CRISPR-Cas systems from viruses to prokaryotic cells and further pointing to the existence of symbioses between bacteriophages and prokaryotes. Interestingly, both horizontal gene transfers and CRISPR defence systems have been associated with a (quasi)Lamarckian mechanisms of inheritance [6,35]. Indeed, these types of acquired genes/spacers depend on the environment in which the bacteria grow. When challenged by viruses, bacteria non-randomly acquire appropriate DNA “novelties” from those “environmental” viruses, allowing bacteria to more quickly respond to that specific virus re-challenge. Therefore, the transferred genes/spacers confer a selective advantage for the growth of the bacteria in that precise environment, exemplifying both the acquired characteristics of Lamarck and the environmentally induced traits of “ecological” views of evolution.

Finally, the viral nucleic acid insertion of non-coding and inactive sequences into prokaryotic cells is an effective cellular tool against viruses that, immunising cells against virus reinfections through a “boomerang response” gives cells a strong selective advantage. That is likely the reason why these specialised systems to fragment and store copies of viral nucleic acid sequences supplanted the mere destruction of viral nucleic acids. Importantly, viral nucleic acid sequences are inserted in a “cell-safe” fashion, far from the cell genome, suggesting that they represent a cellular “strategy” rather than an ongoing threat posed by viruses.

## 3. Viral Nucleic Acid Insertions from the Segregated CRISPR-Cas Sites of Prokaryotes Then Spread into Coding Regions of the Eukaryotic Cell Genome. Are They Harmful or Useful to Eukaryotic Cells? The Hypothesis of Retrotransposon-Guided Human APOBEC Antiviral Activity

### 3.1. The Retrotransposon-Guided APOBEC Enzyme Hypothesis

In humans, members of RNA-guided (APOBEC) mutagenic enzymes have been shown to inactivate viruses, resembling RNA-guided Cas endonucleases [36,37,38] (see Box 2).

Box 2 **Antiviral activity of the APOBEC family.**

The APOBECs belong to the activation-induced cytidine deaminase gene family that originates from the ancestral activation-induced deaminase (*AID*) gene responsible for producing a highly diverse repertoire of antibodies through the somatic hypermutation (SHM) and heavy-chain class-switch recombination (CSR) of immunoglobulins [24,36,39]. A cytidine deaminase domain is responsible for APOBEC cytosine to uracil base modification, finally leading to cytosine to thymidine (C to T) DNA editing [36]. In humans, the APOBEC family consists of 11 different gene products, and some of them have been found to promote extensive (C to T) point mutations on the nascent DNA of some retroviruses during reverse transcription, ultimately leading to virus inactivation (or potentially leading to some immune escape variants) [36,37]. Hence, they may represent the human counterpart of prokaryotic Cas endonucleases. APOBEC expression is upregulated by interferons (IFNs), one of the first/innate immune cell protein responses, and this feature links “innate” IFN production to APOBEC-mediated antiviral activity [36,37]. Interestingly, APOBEC proteins have a well-defined tissue-specific expression [36], suggesting that they were developed to protect distinct tissue cells from different tissue-specific viral infections.

However, APOBECs are active not only against viruses but also against the threat of some endogenous viral sequences, namely the retro-transposable elements [36,37,40,41,42,43,44,45,46,47,48,49], which are part of the human transposons, repetitive DNA sequences that move from one genomic position to another (see Box 3).

Box 3 **Endogenous viral fossils: eukaryotic transposons.**

Transposable elements and transposon-like repetitive elements, originally defined as “junk molecules”, due to their characteristic repetitive sequences, account for almost half of the human genome [50]. The vast majority (about 94%) of human transposable elements are represented by retrotransposons, which duplicate through a RNA copy intermediate by moving from one genomic position to another [41,51]. There are three main kinds of retrotransposons: endogenous retroviruses (ERVs), characterised by long-terminal repeats (LTR), and long and short interspersed nuclear elements without LTR (non-LTR), called LINEs and SINEs, respectively [36,41,50]. ERV and LINE autonomous elements encode specific enzymes to transpose, whereas non-autonomous SINE “parasitic” elements (“cheaters”) use the molecular machinery of the autonomous elements. The majority of ERVs are unable to infect or to retro-transpose due to accumulated mutations; however, during evolution they dispersed numerous IFN-inducible enhancers of innate immunity in mammalian genomes [52]. Therefore, some of these retroviruses function as regulatory sequences able to induce transcription of adjacent IFN-induced genes involved in innate immunity [52], such as the *APOBEC* genes. The most common mammalian LINE, LINE-1 elements, encode 2 open reading frame proteins (ORF1p and ORF2p), which mediate not only the retro-transposition of LINE-1 and SINE, but also the reverse transcription of cellular mRNAs to generate intron-lacking retro-pseudogenes [41]. The ORF2p multifunctional protein with endonuclease and reverse transcriptase activities is responsible for RNA-guided integration of new copies of retrotransposons and retro-pseudogenes into the genome, while ORF1p RNA binding protein possesses a nucleic acid chaperone activity [41]. In addition, ORF1p may also have a LINE-1-translational-repressor activity by binding to its LINE-1 RNA binding site and sterically blocking LINE-1 ribosomal translation and ORF2 protein synthesis (a negative translational feedback loop). In this regard, LINE-1 lacking the ORF1p coding sequence has been shown to strongly increase ORF2p-mediated Alu retro-transposition (see Figure 2A in [40]). It is thus possible that, similarly to the Cascade complex in CRIPR-Cas systems (see above), in (stressful) conditions inducing an “excess” of LINE-1 transcription, ORF1p redistribution towards the surplus of LINE-1 hyper-transcribed elements (and consequently its sequestration) would relieve translational repression, allowing a rapid ORF2p translation and consequently an increase in transposon transposition. Regardless the molecular mechanism of transposition induction, the majority of the several hundred thousand copies of LINE-1 are truncated and transpositionally inactive [50]. Among SINE, Alu sequences are the most successful elements in the human genome; however, they do not encode proteins, and the vast majority are transpositionally inactive elements [41,50]. They are derived from the evolutionarily conserved 7SL RNA viral sequence, a component of the signal recognition particle involved in protein secretion [41,50,53]. The different Alu subfamily members contain two (left and right) 7SL-derived Alu domains and a 3′ flanking unique genomic sequence, which characterises each Alu in its singularity [41,50,53,54].

Retrotransposons are sequences of viral origin which, unlike spacers in CRISPR systems, are considered parasitic DNA sequences dispersed into the eukaryotic genome, whose activity must be tightly controlled to maintain host genome integrity. Indeed, ERV human retro-elements contain viral DNA sequences which codes for viral proteins with potential infectivity, and non-LTR elements can potentially damage host genes during their transposition [36,41,50,55]. However, some transposons behave like “tools for host genome engineering” that are successfully involved in both immune systems and natural genome editing mechanisms [33,56].

In humans, the variety of APOBEC proteins is thought to be crucial in countering the genotoxic threat generated by endogenous retro-elements [36,40,41]. Indeed, the expansion of the APOBEC family during primate evolution coincides with a decrease in transposon activity [36]. However, the elevated genotoxic activity of APOBECs is also well known [36], curiously suggesting that the APOBEC response could be even more dangerous than transposon activation.

The presence of fossil forms of previously inserted viral sequences with accumulated mutations raises the question of why sophisticated eukaryotic cells possess such a huge amount of apparently useless and potentially harmful viral DNA. Are transposons intrinsically selfish as genes are hypothesised to be? Or have retrotransposons developed other functions in addition to retro-transposition?

Importantly, unlike virus and ERV inactivation, APOBEC inhibition of LINE and SINE retro-transposition is usually independent of their mutagenic function [40,42,43,44,45,46,47,48,49]. Rather, it seems to be mediated by binding and sequestration of retrotransposons away from their genomic integration target sites [40,42], which express the consensus sequences for retro-transposition [41]. Finally, both RNA-guided Cas endonucleases and APOBEC mutagenic enzymes may represent immune effectors which can bind RNA nucleic acid fragments of viral origin, repeat–spacer sequences and retro-transposable elements, respectively. Indeed, APOBECs show specificity for individual LINE-1 and Alu transposable elements, and distinct binding sites between some retrotransposons and specific APOBECs have been described [38,40,42,43,49,54], raising the possibility that some retro-transposable elements, like repeat–spacers in CRISPR-Cas systems, could represent RNA-guides for APOBECs in human eukaryotic cells.

### 3.2. How Have Endogenous Viral Sequences Spread into the Coding Regions of the Eukaryotic Cell Genome?

Unlike prokaryotic spacers, which are segregated into CRISPR-Cas immune organs, eukaryotic transposons spread into the eukaryotic genome. Indeed, Alu sequences, the most successful retro-elements in humans, are mainly present in “sensitive” intronic and intergenic DNA regions [57]. How and why have transposable elements been integrated into transcribed regions of the eukaryotic cell genome?

It has already been suggested with regard to CRISPR-Cas systems that viral nucleic acid integration into the cell genome gives the cell a survival advantage and likely represents a cellular strategy to control the viral threat. Can this also be true in the case of hypothetical retrotransposon-APOBEC systems?

During the first viral challenge, eukaryotic cells transcribe specific genes in response to the viral threat. It is conceivable that the viral fragment insertion could occur more easily in unfolded open state regions of the chromatin with single-stranded DNA (ssDNA), i.e., accessible transcribed DNA regions, during viral infection. Random viral fragment insertions usually produce disruption of the transcribed gene and cell death, which also stops the spreading of the virus. However, if the viral fragment insertions maintain the integrity of the proteins translated from the “infected” genes, the cell can survive. Notably, this can be achieved when the step sequence for viral integration is reversed after transcription by a transcript processing event that completely removes viral fragment insertions, a mechanism that is reminiscent of the intron-exon splicing process developed in eukaryotic cells. From this standpoint, eukaryotic RNA splicing would represent the reverse reaction of the viral nucleic acid insertion and would be capable of restoring the original gene sequence without the viral sequence insertions. Finally, these intron-like viral insertions would fragment genes without affecting the structure and function of the translated proteins. This could be the origin of the endogenous viral sequence spread into the coding regions of the eukaryotic cell genome. However, since this type of gene fragmentation has been positively selected and generated eukaryotic cells, it must have been advantageous to eukaryotic cell survival.

### 3.3. Antiviral Activity of Retrotransposon-Guided APOBECs

Drawing a comparison with CRISPR-Cas systems raises the question of whether this intron-like viral nucleic acid insertion is functional against the next viral entry. Importantly, a new threat of previously experienced viruses will activate the same sequential responses and transcriptions of the same genes transcribed during the first infection. However, in this second response, in addition to the DNA coding sequences, the inserted viral nucleic acid (intron-like) fragments will also be transcribed. As in the case of repeat-spacers for Cas endonucleases, the ability of viral RNA fragments (retrotransposons) to bind to APOBEC mutagenic enzymes (if simultaneously transcribed) would quickly guide APOBECs against viral sequences. Indeed, APOBEC3G has been shown to associate with 7SL RNA and Alu elements [38,42,54]. A conserved motif of APOBEC3G is important for the interaction with these RNA viral sequences and association with them has been shown to mediate the targeting of antiviral cytidine deaminases against retroviral complexes [38,54], suggesting that some Alu/Alu-like elements could represent RNA-drivers of APOBECs against exogenous retroviruses. The conserved repetitive sequences on Alu domains [50,54], like repeats for Cas endonucleases, would allow their binding with APOBEC3G, while unique 3′ sequences [41], like spacers, would ensure target specificity.

The cell-safe viral DNA fragment integration into eukaryotic cell genes would be a useful mechanism to protect the cells from subsequent reinfections. Therefore, the ability to store a copy of viral fragments (transposons) within the intronic regions would give the cell an advantage through a “boomerang response” similar to that described for CRIPR-Cas systems. Indeed, the possibility that introns might be captured sequences of viral origin (e.g., viroids or viroid-like RNAs) was first proposed by Diener [58]. Finally, the viral nucleic acid dispersion into intronic and intergenic regions was likely selected because it was helpful against virus reinfection, becoming a cell “strategy” to counter new viral threats.

### 3.4. The Eukaryotic Gene Development Hypothesis

It is likely that this mechanism of viral fragment insertion occurred initially in transcribed poly-cistronic prokaryotic genes and was the basis for the development of eukaryotic introns, but possibly also of eukaryotic promoter and enhancer regions. Indeed, several response elements for transcription factors of eukaryotic introns, promoters and enhancers are repetitive sequences embedded in Alu elements [59], all elements of viral origin disseminated within genic and intergenic regions of the eukaryotic cell genome. Notably, some viral nucleoproteins have an intrinsic ability to associate with their viral nucleic acid sequences. It is thus possible that during viral infections, some viral nucleoproteins could bind viral repetitive elements previously inserted upstream from the gene start site and in inter-cistronic regions. These viral sequences could therefore function as specific cell sensors for viral products. In fact, it can be hypothesised that, like eukaryotic transcription factors, the steric hindrances created by some viral nucleoproteins could help to keep the gene “open”, enhancing the transactivation of previously “infected” genes with intronic (anti)viral sequences. In the course of evolution, we can observe a progressive reduction in the time required to generate a response to counter external threats. Indeed, response speed is fundamental to survival. This mechanism of viral insertion would yield both an enhanced and more rapid response to viral threats, and this could account for its positive selection. From this point of view, retro-pseudogenes inserted into the genome by the LINE-1 machinery would represent the first step in gene duplication and copy number variation just before the insertion of the promoter and intron regions by exogenous or endogenous viral fragment insertions.

### 3.5. Cell Integration of Viral Elements: The Richness of Foreigner-Migrant “Cultures” Following Unproductive Struggles

As mentioned above, there is similarity of function between some viral nucleoproteins and transcription factors, suggesting that some eukaryotic transcription factors might derive from the editing of some viral nucleoprotein genes inserted into the cell genome. In this regard, there is evidence indicating that some transcription factors derive from the “domestication” of viral transposases [60]. Transposases are viral nucleoproteins that mediate the insertion of the viral nucleic acid from which they are encoded (for example ORF2p for LINE-1 elements). Domestication of viral proteins occurs when viral coding sequences are “exapted” to generate functional host proteins [61,62]. But what could be the advantage that led to integration and editing of viral coding sequences?

There is no reason to synthesise viral-like proteins in the absence of the virus; however, the endogenous synthesis of viral-like proteins makes sense if linked to viral infection. Such synthesis is an amplifying mechanism of viral perception at low loads, eventually increasing the speed of cell response, which was likely the reason for its initial positive selection. Indeed, several viral proteins have been co-opted by their cell host to restrict viral infection [32,33]. Among the cell proteins able to restrict viral spread, APOBEC family members have a crucial role. Their intrinsic ability to bind to viral sequences suggests that they might have developed from viral nucleoproteins, which are different from those generating transcription factors. In this regard, there is evidence that deaminase domains, determining functional characteristics of the APOBEC proteins, have been acquired by eukaryotes from bacterial toxin systems [63], pointing to an indirect mechanism of viral domestication mediated by a bacterial intermediary.

Finally, cell-domesticated viral coding sequences and proteins, as well as viral non-coding sequences would likely have been, at least initially, exploited by host cells as a boomerang against the same viruses from which they derive. However, the endogenous viral sequences, which represent almost half of the cell genome, were likely inserted and domesticated against ancient viruses that no longer exist. For this reason, the existence of this enormous and seemingly useless or even dangerous graveyard of viral zombies within the sophisticated eukaryotic cell genome remains unclear. Have they developed any other functions over time that may be of use even today?

It is known that *APOBEC* genes evolved in primates [36], concomitantly with LINE-1 elements [41], over 100 million years ago from the *AID* gene, which appeared more than 500 million years ago [36]. On the other hand, both Alu amplification [41] and *APOBEC* gene expansion [36] occurred between 55 and 35 million years ago; based on their time of expansion, Alu should have binding sites for the “younger” APOBECs, while LINEs should have binding sites for the ones that evolved earlier. Taking advantage of co-evolution of the APOBEC family members and some retrotransposons in primates, it is possible to hypothesise that the expanding range of mutagenic enzymes were tools developed to counter the burst of several types of ancient (tissue-specific) retroviruses [51]. Moreover, the cellular integration of Alu and LINE subfamily retro-elements, for which APOBECs have conserved binding domains, may be the result of retrovirus “domestication”. Importantly, these ancient retro-elements, acting in concert with APOBECs, would still be useful to counter newly evolved viruses, such as HIV [38,54]. From this standpoint, even the integration of HIV fragments and defective HIV-1 provirus producing novel HIV protein-coding RNA transcripts [64] could represent not only a “Trojan Horse” reservoir of HIV, but also products of virus domestication and new biotechnological tools to combat HIV itself and/or future viruses. Similarly, plasmid-like unintegrated circular HIV DNAs [65] might represent tools to spread either infective or domesticated HIV DNAs. Hence, it is possible to imagine a new generation of vaccines that aim to insert specific HIV-engineered transposons with an appropriate unique sequence recognising conserved HIV sequences and conserved repeats for APOBEC binding. To target them into genes that are transcribed early during viral entry in CD4 positive cells, engineered transposons could be loaded into HIV-like capsids/exosomes with affinity for CD4 molecules and able to produce cell membrane stresses typical of viral infection in CD4 positive cells. This type of insertion would be functional because it would first allow the integration of the engineered transposons into specific genes of HIV CD4 target cells during vaccination and their subsequent intronic transcription in the case of HIV virus infection.

In conclusion, natural selection, following countless unproductive cell-virus struggles, may have led to a cell-virus agreement, a symbiosis, in which old viruses became fundamental components of the immune cell organ at the cell’s disposal against newly evolved viral threats. Similarly, the microbial communities in symbiosis with their host organism or their host-integrated bacterial sequences are integral to numerous host processes including immune system development and responses [11,16,17,26,63].

In this scenario, it can be observed that natural selection at different levels of biological organisation, promotes mechanisms of microbial “education” and “integration”, rather than of domestication and exploitation. The splicing mechanism described above also appears to be in line with this hypothesis of viral tool integration. Indeed, there are several lines of evidence suggesting that the catalytic centre of the spliceosome is composed of RNA, i.e., it is a ribozyme [66], a small catalytic RNA found in a number of RNA virus genomes and viroids [2,3,58,67].

Interestingly, through these mechanisms, the viral migrants became relatively “sedentary” viruses, still “alive” in the cell environment, raising the possibility that cells represent a community generated by sequential viral integrations not only of versatile operon modules but also of targeting repetitive sequences that, by binding to conserved protein domains, perform regulatory functions.

## 4. Exaptation of Human Retrotransposon-APOBEC Systems: From Antiviral Activity to Chronic Stress-Induced Site-Specific Genome Editing

### 4.1. Transposon- and APOBEC-Mediated Genome Editing Mechanisms: A Possible Link with Environmental Stress Conditions

Both CRISPR-Cas and retrotransposon-APOBEC systems would appear to be cell immune systems developed to counter viruses, which both use viral nucleic acid fragments to guide immune effector enzymes in prokaryotic and eukaryotic cells, respectively. However, unlike Cas endonucleases, APOBECs mediate viral mutagenesis, suggesting that in eukaryotic cells, viral genome editing was positively selected over its fragmentation. A new cell mechanism is positively selected only if it produces improvements in cell fitness, so new adaptive abilities should be expected in retrotransposon-guided APOBEC systems. Notably, unlike genome-dispersed transposons, segregated CRISPR systems are protective against “viral toxicity”, yet they limit viral nucleic acid insertion, and thus cell variability. Interestingly, under stress conditions, an archaea species has been observed to activate a primordial form of sexual interaction, a species-specific horizontal form of nucleic acid transmission [68]. This primitive sexual mechanism might have been selected to compensate for a reduction in viral operon module insertion (variability), and notably, it is activated under stress-inducing environmental (ultraviolet rays) conditions, i.e., when an increase in genetic variability is functional to solve the problems imposed by new environmental conditions.

Interestingly, APOBEC mutagenic functions are directed not only against viral sequences, but also against human endogenous (viral and non-viral) DNA, a feature responsible for both human evolution and human cancer generation [36,69,70]. Indeed, some APOBEC members (e.g., antiviral APOBEC3G) have been shown to contribute to the human genome evolution through site-directed mutations of transcribed regions and regulatory elements [69], while others (e.g., APOBEC3B) have been associated with carcinogenesis [36,70], suggesting an exaptation of their original anti-viral mechanism to a genome editing mechanism. Similar effects have been described for transposons [23,71,72], indicating that both transposons and APOBECs have developed new beneficial as well as harmful functions through genome editing mechanisms. Indeed, roles of the CRISPR-Cas machinery in directing processes other than immune functions, such as endogenous gene regulation, genome evolution by self-targeting genome editing, and CRISPR-guided transposition, have been observed [33,73,74,75,76], providing additional support for the idea that retrotransposon-guided mutagenic enzymes might represent the eukaryotic versions of prokaryotic CRISPR-Cas systems. Indeed, the manner of prokaryotic CRISPR/RNA-guided transpositions [74,75,76] is reminiscent of human/eukaryotic LINE-1 transposition mediated by RNA-guided ORF2 multidomain (“fusion”) protein [41]; a transposition mechanism that preceded that of non-autonomous SINE elements. The functional similarities of CRISPR-Cas to retrotransposon-APOBEC systems imply their evolutionary “convergent” relationship, although at different (prokaryotic and eukaryotic) levels of biological organisation. In any case, positive selection of mutagenic genome editing over nucleic acid fragmentation mechanisms must have improved eukaryotic cell fitness.

Indeed, dynamic DNA editing processes produce a series of events including gene point mutations, gene duplications and chromosomal aberrations that ultimately lead to novel phenotypic traits (phenotypic plasticity) and enhanced ability to face stressful environments [9,11,17,23]. However, these events are crucial not only to organism but also to cancer adaptation and perpetuation, which ultimately leads to organism death. Hence, we can observe that similar effects produce completely different outcomes! Intriguingly, DNA editing events are clustered non-randomly in localised DNA areas of elevated recombination activity, the so-called hotspot genomic regions, in which single nucleotide polymorphisms (SNP), gene copy number variations (CNV) and chromosomal translocations are generated. Local increases in mutation density (occurring even in a single generation) produce heterogeneity/plasticity at different levels of organisation (cell and organism) and have been associated with primate evolution as well as with human cancers [5,69,70], suggesting that both involve common mechanisms of non-random site-specific genome editing. In this regard, in prostate cancer cells, genotoxic stress has been shown to induce non-random chromosomal translocations mediated by upregulation of LINE-1-encoded ORF2 protein and AID [77], highlighting the importance of transposons and AID/APOBECs as agents of genome remodelling and cancer development in stressful environmental conditions. Moreover, it has been revealed that transposon insertions are involved in both leukaemic and non-leukaemic chromosomal translocations [71,78,79,80], suggesting that “transposon-driven” translocations are not themselves sufficient for cell malignant transformation. Similarly, there is evidence of somatic mutations in healthy human tissues that resemble those observed in cancer cells [81], suggesting that, in some cases, somatic mutations and chromosomal translocations can be neutral or even beneficial. In this regard, chromosomal translocations are responsible not only for cancer development [71,77], but also for “Robertsonian speciation” [20], suggesting that cancer development or cell/organism adaptation might also be related to the environmental context. However, different environmental (stress) conditions can not only select and promote different biological phenotypes but also activate different transposon expression [5,23,71,72]. In this regard, the stress-response hypothesis of Barbara McClintock proposed that the expression of mobile elements was a cell genomic reaction to stressful environmental conditions [82]. However, what might the origin of specific transposon activation be?

### 4.2. Stress-Induced Transposon Mobilization: The Intronic Origin Hypothesis

It is known that major environmental changes can affect transposon silencing and subsequent transposon activity has been associated with genetic variability, genomic instability and cancer [23,71,72]. For these reasons, transposons have been defined as parasitic elements with their own autonomous ability to reactivate under stress conditions and to threaten genome integrity. However, the molecular links between environmental stress conditions and transposon upregulation or de-repression are elusive. There is no clear evidence of a specific mechanism for transposon transcription mediated by stress-induced regulatory proteins. On the other hand, we now know that the majority of transposons are present in intronic and regulatory regions. The vast majority (about 90%) of human genes contain transposable elements in their introns and more than a million transposons are present in the introns of the human genome [71,83]. Since the regulatory regions are not transcribed, a main source of transposable elements might be the introns of genes induced to transcription in response to stress conditions. In this regard, many stable intron products have been discovered [84] (see Box 4).

Box 4 **Non-coding stable introns after splicing.**

It has been well documented that after the splicing of transcribed genes, some introns generate circular molecules, lariat intermediates and lariat intron products [84]. These intron-derived products present internal phosphodiester bonds (branchpoint), which protect them from digestion by exoribonucleases [84]. Indeed, hundreds of stable lariat intronic RNAs have been found in the cytoplasm of different vertebrate cells, including human cells [85]. These stable transcripts derive from short introns coming from genes of widely different cellular functions [85] and their regulation is tightly controlled, as it would be expected from functional RNA species [86]. Indeed, during inflammatory stress conditions different stable intronic transcripts are differently regulated, even when they are derived from the same locus, suggesting that they represent a reservoir of RNAs with yet unknown functions [86]. Lariat-introns are subsequently cleaved to linear form by the lariat-debranching enzyme, DBR1, before their digestion or function. Interestingly, retro-element transposition requires the lariat-debranching enzyme [87,88], suggesting that retrotransposon intermediates are contained in some lariat-introns and the lariat-debranching process is necessary for their retro-transposition.

Notably, stress-induced transposon transcripts do not produce a proportional increase in the transposition rate [23], suggesting that intronic transposons might have developed functions other than transposition, possibly activated by cells under environmental stress conditions. Although the biological significance of cytoplasmic stable intronic RNAs remains obscure, one simple hypothesis is that some cytoplasmic lariat intronic RNAs may interact directly with RNA-binding proteins such as RNA-guided APOBEC enzymes. Finally, I propose that, under stress conditions, at least part of the transposons may come from the introns of genes transcribed in response to stressful environmental conditions. However, what do the environment and environmental stress mean for a cell?

### 4.3. Molecular Concepts of the Cellular Environment and Cell Environmental Stress Conditions: An “Ecological” View of Cells and Tissues

Excluding physical agents, the environment for a cell in an organism is mainly represented by cell “exogenous” molecules that come into contact and sterically interact with molecules of the cell, hereafter exogenous environmental molecules. Exogenous molecules that come in close proximity to a cell and do not sterically interact with its components are “invisible” to the cell, and since they do not interfere with cellular functions, they are not directly part of the cell environment. Notably, exogenous environmental molecules can be tissue specific, since they may or may not interact with molecules expressed by a specific tissue cell, depending on three-dimensional structures of both exogenous molecules and cellular proteins. In addition, their binding to cellular structures can be not only tissue specific but also dependent on a specific protein polymorphism and therefore individual specific. Exogenous environmental molecules can be autonomous molecules but also molecules present on the surface of neighbouring cells or extracellular components. Therefore, a cell interacting through its surface molecules with neighbouring cells constitutes an environment for those neighbouring cells. Similar concepts can be transferred to organism interactions and to genomic and epigenetic interactions, in which the epigenetic factors would represent environment for the genome.

In normal environmental conditions, exogenous environmental molecules fluctuate around normal concentration ranges through assumption, storage and release (homeostatic) mechanisms, which tend to limit their fluctuations and maintain optimal concentration ranges through negative feedback loops. Likewise, cellular proteins fluctuate around optimal concentration ranges as a function of environmental conditions. However, when specific cell components interact with new, never-experienced exogenous molecules, those molecules can interfere with cell biological functions, generating tissue-specific cell perturbation and stress. Such molecules are usually defined as pollutants or stressors and they can be biotic or abiotic. Another important consideration is the concentration of exogenous environmental molecules. There are normal (tissue specific and individual specific) fluctuations of molecule concentrations to which the cell is able to respond through homeostatic mechanisms, and abnormal ones (out of the normal range), either excessive or insufficient, which produce cell stress. In this regard, the concentration of exogenous molecules in a specific tissue cell may depend on the organ of entrance in the organism. For example, air pollutants interact at high doses with pneumocytes, while food pollutants interact at high doses with enterocytes and hepatocytes, which is probably why smokers are more likely to develop lung cancer than other malignancies. Notably, high concentrations of molecules (pollutants or non-pollutants) induce low-affinity (non-specific) bindings to cellular molecules, therefore new cell perturbations and stress, which do not occur at low concentrations. However, intermolecular bindings mediated by weak bonds depend on concentrations of exogenous environmental molecules but also on concentration and polymorphism/structure of cellular proteins, because both concentrations and affinities determine the probability of their interaction. Finally, both structures and concentrations of environmental pollutants and cell molecules can cause significant changes in the cell environment and cell stress level that may be either temporary or chronic. Notably, when a specific tissue is “environmentally” stressed, it responds to environmental perturbation with a plastic adaptation and a “niche construction” that may affect other tissues interacting with it, ultimately generating a cascade of events involving the whole organism.

### 4.4. Cellular Homeostatic Mechanisms Regulate Optimal Cellular Protein Concentrations in Normal Environmental Fluctuations

**Figure 2:**
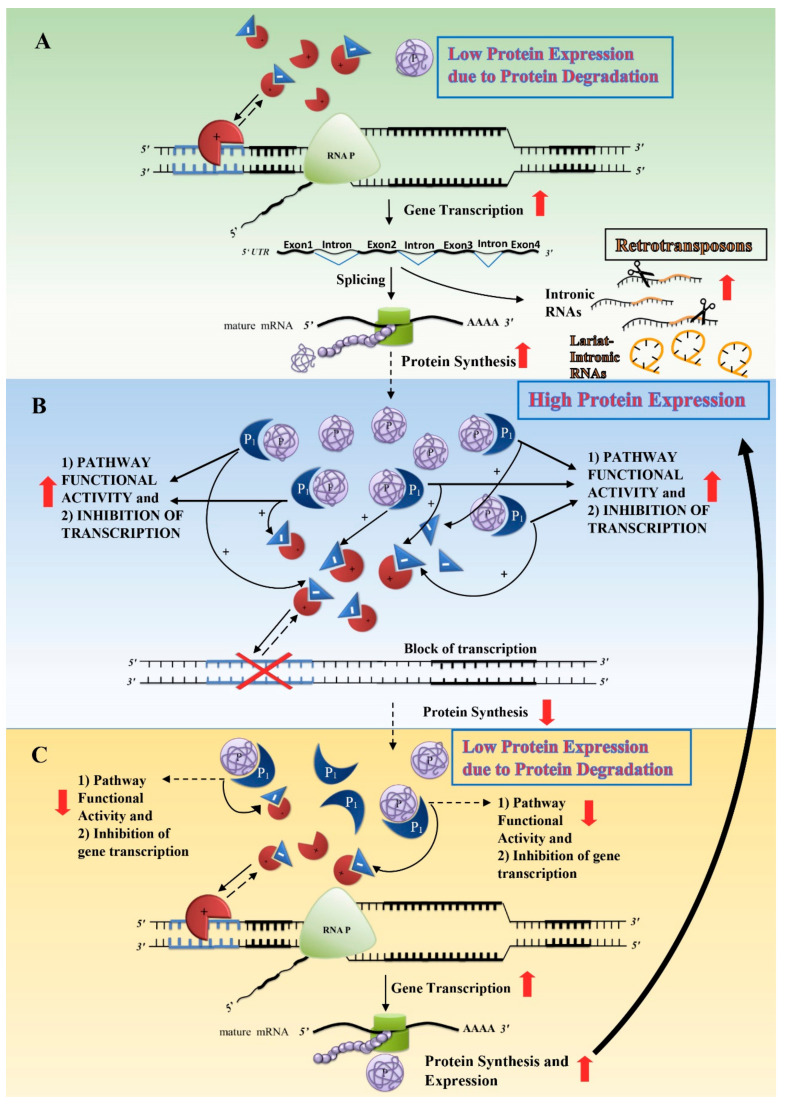
Cellular homeostatic mechanisms regulate optimal cellular protein concentrations in normal environmental fluctuations. Sequential steps (**A**–**C**) of cyclic protein synthesis and protein fluctuations. The example in the figure depicts a homeostatic mechanism involved in the protein expression of a signal protein at a transcriptional level (for reference, see the text). RNA P: RNA polymerase; P/P1: protein/protein 1; 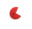: transcription factor; 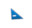: repressor; 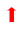: increase; 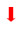: decrease.

Cells and organisms have developed specialised homeostatic systems able to fine tune the amount of protein in cells as well as the number of cells within an organ according to the environmental context. There is an optimal range of expression for each cell protein and a protein excess or deficiency produces cell dysfunction and cell stress. Changes in environmental conditions produce cell perturbations and cell responses seek a new balance (adaptation). Dynamic balances through homeostatic mechanisms regulate optimal protein expressions as a function of environmental conditions. Homeostatic responses are usually epigenetic mechanisms, which, operating through transcription factors, repressors and enzymes, modulate protein expression and stability, ultimately regulating their amount, persistence and functionality at transcriptional and/or post-transcriptional levels. Cell homeostatic mechanisms adjust the protein amount, function and stability according to the cell functional needs, typically through negative feedback regulatory loops. For example, Figure 2A–C shows a homeostatic mechanism involved in the protein expression of a signal protein at a transcriptional level.

When a protein (P) is expressed at low levels with respect to the cell’s functional needs (Figure 2A), the transcription factor (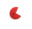) that regulates its transcription is free to induce its gene transcription. The subsequent translation into proteins increases the protein amount and, by binding with a second protein (P1), the pathway functional activity in which the protein (P) is involved (see Figure 2B). To fine-tune protein expression, cells have developed negative feedback mechanisms through repressors (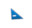) able to inhibit the activity of transcription factors (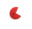): the higher the expression of the protein (P) bound to its partner protein (P1) along their signal pathway, the higher the gene repression (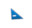), which inhibits gene transcription. Finally, lowering protein synthesis, protein degradation produces both low levels of protein (P) and repressors (Figure 2C), similar to the initial condition (Figure 2A). The cycle restarts and the amount of protein (P) fluctuates around the optimal needs for cell functionality according to the environmental conditions. Usually, cell epigenetic homeostatic responses counteract normal environmental fluctuations through negative feedback loops that tend to reduce external environmental molecular changes around a normal fluctuating range. Normal environmental fluctuations induce either an increase or a decrease in gene transcription and protein expression and stability. However, a major problem arises when a new environmental context induces cell homeostatic responses which, though reaching their maximum activity, are not sufficient to meet the new cell needs. This occurs when the demand surpasses the potential ability to synthesise and stabilize a specific protein, i.e., the cell does not find the solution/balance among the epigenetic mechanisms. If the new environmental condition is temporary, it may produce a temporary cell stress; however, when the environmental changes are persistent and/or increasing, chronically stressed cells have to activate different mechanisms in order to survive.

### 4.5. Putative Cellular Mechanisms of Eukaryotic Non-Random Genome Editing in Response to Chronic Stress Conditions: Chronic Stress-Induced Gene Duplication and Site-Specific Mutagenesis

I have already described how cell homeostatic responses counteract normal environmental fluctuations and a new “epigenetic” balance is rapidly attained (see Figure 2). However, in the case of never-experienced and chronic environmental conditions, for which the cell does not possess an “epigenetic” solution, i.e., the protein requirements exceed the homeostatic possibilities, the cell homeostatic responses are chronically pushed to their limits.

As already suggested, chronic cell stress can be generated by new exogenous molecules that permanently interfere with cellular components or when the cell is constantly exposed to abnormal (either excessive or insufficient) concentrations of “known” exogenous molecules. Persistent environmental stressors continue to perturb cell functions until cell adaptation or cell death occurs. But what is the sequence of events that could lead to cell adaptation in chronic stress conditions?

Sudden and intense stress conditions (e.g., under volcanic eruptions, earthquakes or meteorite impacts) may not leave enough time for cell/organism adaptation, leading to mass extinctions [14,15], whereas chronic environmental conditions that originate from a progressive deviation from normal environmental fluctuations would first induce cell epigenetic homeostatic mechanisms. It is likely that epigenetic mechanisms of adaptation are trained by progressive environmental changes. However, epigenetic homeostatic mechanisms likely have a threshold of tolerance beyond which the final products of cell epigenetic responses, specific protein concentration, functionality and stability, would not be able to provide an effective response to the new environmental conditions. While the minimal needs for proteins can be easily met by blocking gene transcription, the optimal amount of protein and protein function cannot be attained in the case of an excessive demand, which exceeds the cell’s epigenetic capabilities. In chronic stress conditions, some genes may be switched off by promoter methylation and others may be permanently switched on by promoter de-methylation and chronically transcribed, “hyper-transcribed”. However, when “hyper-transcribed” genes do not produce sufficient amounts of proteins to meet new cell needs, the genes are unable to provide an adequate response to the new environmental conditions. More generally, we can say that hyper-transcribed genes are genes which are inadequate for the environmental context.

In these situations, gene duplications and/or gene editing (“genetic plasticity”), in search of more effective proteins, are two possible independent ways to achieve a new balance. Finally, CNV and SNP of hyper-transcribed genes may allow cells to surgically explore a new fitness scenario, which increases their adaptability to stressful environmental conditions. In a changeable environment, like that on Earth, randomly emerging mechanisms able to produce these effects were likely positively selected. However, what mechanisms could make adaptation of hyper-transcribed genes possible?

A first possible response to excessive demand for a specific protein is gene duplication. To synthesise eukaryotic proteins, first gene transcription, then splicing, and finally translation are sequentially induced. The “rate determining steps” in these “reactions” are likely the splicing event and the base pairing between codon and tRNA-anticodon carrying an amino acid during gene translation. In the case of hyper-transcribed genes, an overflowing/excess of mature and “dis-spliced” (or badly spliced) transcripts waiting for ribosomal protein synthesis is therefore expected. It is possible that, during stress conditions, the excess of multiple-sized transcripts from hyper-transcribed genes might favour the interaction of intronic “dis-spliced” transcripts with promoter/enhancer regions of hyper-transcribed genes themselves (inhibiting their transcription) and/or mRNA (spliced or dis-spliced) transcripts with stress-induced LINE-1-encoded ORF2 multifunctional proteins (leading to gene copy number variation). These hypotheses are supported by the observations that highly expressed housekeeping genes have both RNA-mediated negative feedback loops (e.g., in ubiquitin overexpression [89]) and high copy numbers of retro-pseudogenes [41]. Indeed, interaction of multiple-sized transcripts with ORF2p may generate retro-pseudogene or fragmented gene insertions of hyper-transcribed genes in the accessible chromatin regions of the hyper-transcribed genes themselves. As suggested above, in this scenario, retro-pseudogenes (and gene fragments) would represent the first step in gene duplication and CNV, just before the “viral” insertion of new promoters, enhancers and introns. In line with this hypothesis, there is evidence that salivary amylase gene copy number in the human genome is positively correlated with starch diets, indicating that gene copy number are plastically tailored to meet specific environmental needs [90]. However, the new gene copy could diverge from the original gene because of its fragmentation and/or as a consequence of the different promoter and intron insertions caused by new virus infections and/or the new transposon insertions, i.e., by cell environmental conditions.

Gene editing and generation of SNP in hyper-transcribed/inadequate genes represent another way with which cells can achieve a new cell balance in response to chronic stress conditions. This mechanism explores different gene point mutations to be tested in the new environmental context and those providing the highest level of cell fitness and survival capability will be inherited. What could the mechanism of surgical selection for editing an inadequate gene be?

**Figure 3:**
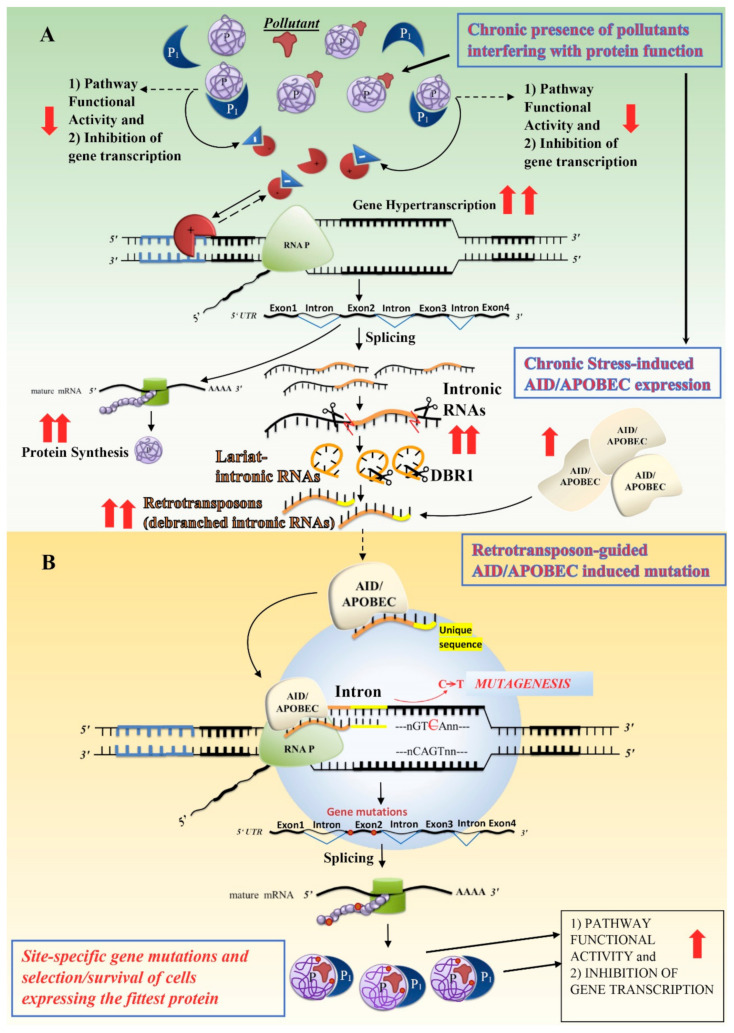
Putative cellular mechanism of eukaryotic genome editing in response to chronic stress conditions. Sequential steps of gene hyper-transcription (**A**) and gene mutagenesis (**B**) induced by a pollutant binding to a protein. An example depicting chronic stress-induced site-specific mutagenesis mediated by APOBEC enzymes. (for reference, see the text). RNA P: RNA polymerase; P/P1: protein/protein 1; 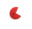: transcription factor; 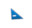: repressor; 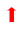: increase; 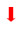: decrease; DBR1: lariat debranching enzyme.

Figure 3A shows the case in which a new biotic or abiotic pollutant chronically binds and interferes with a signal protein (P), affecting its binding with a second protein (P1) along its signal pathway.

Both the protein (P) functional activity and its repression are inhibited by the pollutant and homeostatic epigenetic responses induce upregulation of gene transcription (and post-transcriptional protein stabilization and functionality). An increase in protein synthesis is functional because it compensates for the lack of protein function induced by the binding with the pollutant (a negative feedback loop that reduces the pollutant-driven cell perturbation, but also favours the further binding with the pollutant). However, hyper-transcription of the gene produces not only an increase in its mRNA for protein synthesis, but also in the intronic transposons present (if any) in the hyper-transcribed gene. In the case of sustained transcription, such as in chronic stress conditions, transposon RNAs from hyper-transcribed intronic regions would be accumulated within the cells. Indeed, many stable lariat intronic RNAs derived from hundreds of different genes have been shown to be exported to the cytoplasm. They are short, mostly 100–500 nucleotides in length, and they are expressed at 10% or more of their cognate mRNA level [85]. Importantly, stress conditions can also induce APOBEC upregulation, and I have already suggested that some transcribed retrotransposons can bind and guide these mutagenic enzymes. It is therefore possible that stable retrotransposon transcripts (after the DBR1-mediated lariat-debranching process) might guide APOBECs towards the genomic introns from which they were transcribed. When the chromatin of a gene is in an open state, such as during continuous gene (hyper)transcription, the specific intronic site would be readily accessible for editing.

This mechanism would foster the generation of mutations without DNA duplication on both gene alleles codifying for the protein involved in the binding with the pollutant, leaving the rest of the genome intact (Figure 3B). I have already suggested that the pollutant structure can bind to a specific tissue protein. Therefore, after chronic binding with pollutants, cells of specific tissues can be induced to randomly generate slightly different mutants (genetic plasticity) of hyper-transcribed genes. Although this mechanism could potentially occur in virtually all cells, it is likely that only long-lived cells, such as adult somatic stem cells or memory cells, could survive and respond to chronic environmental stressors. However, tissue-specific stem cells might not express some tissue-specific proteins, therefore some mature tissue cells might reactivate the stem cell program (as in memory cells). Alternatively, stem cell proliferation and differentiation induced by tissue cell death of mature cells might lead to progenitor cells both with stem cell renewal capacity and expressing tissue-specific proteins. Regardless the type of cells and the mechanism, among the cell mutants expressing different protein variants (phenotypic plasticity), those bearing gene mutations which provide high cell fitness in the new organism environment would survive and expand, ultimately yielding a new gene version “inheritable” by tissue progeny (genetic assimilation). In line with this hypothesis, recent evidence has revealed intra-organismal and intra-tissue genetic mutations (genetic plasticity) in healthy individuals [91]. Indeed, it has been observed not only mosaicisms of both SNP [91] and CNV [92,93] within and among normal tissues of the same individual but also tissue-specific and age-related mutation profiles associated with gene transcription in healthy tissues [81]. In addition, tissue-specific translocations, pathological or non-pathological, can represent a third level of mosaicism and an expression of chromosomal plasticity.

As shown in Figure 3B, a protein mutant (P) able to restore binding with (P1) in the presence of a pollutant is a good solution for the survival of both tissue cells and the organism in which the cells reside. Interestingly, this hypothesised mechanism of gene editing mediated by APOBECs would produce different protein repertoires (phenotypic plasticity) and binding affinities for the pollutant, and eventually the mutated protein would somehow wrap around the pollutant, by binding it with higher affinity than the original protein. This process strongly resembles the somatic hypermutation (SHM) of immunoglobulins (Igs) mediated by AID, the ancestor of the APOBEC family, during commitment to B cell memory [24,36,39]. It is well known that generation of long-lived memory cells is characterised by new gene expressions mediated by epigenetic modifications that drive “naïve” cells to differentiate into long-lived cells [94]. If this is the case also in other tissues, in general, new tissue mutants might derive from mature cells that reactivate the stem cell program rather than from stem cell differentiation. Altogether, these mechanisms produce both epigenetic and genetic variations that yield a range of environmentally induced phenotypes (phenotypic plasticity). Finally, environmental selection of the fittest phenotype would ultimately fix (environmentally independent) the acquired gene versions into the genome, leading to genetic assimilation of environmentally induced characteristics.

Is there any molecular evidence of this putative cell mechanism in human eukaryotic cells?

### 4.6. Learning from the Immune System: The Example of AID-Mediated Immunoglobulin Recombination

As mentioned above, the mechanism model that I have proposed resembles SHM of Igs, a process which is usually coupled to heavy-chain class-switch recombination (CSR) of Ig locus, both of which are induced by AID [9,10,24,39,95,96] (see Box 5).

Box 5 **Somatic hypermutation of immunoglobulins and heavy-chain class-switch recombination.**

SHM and CSR represent two pivotal processes in the immune system able to edit Ig genes in a way that adapts them to a new biotic pollutant (antigen) through targeted mutagenesis. During these processes, the Ig gene undergoes: (1) point mutations in the variable, antigen-binding coding sequences, which change antibody binding affinity for the antigen and (2) a class switch from IgM to IgG, IgE, or IgA, which changes the antibody effector function. These processes start when the binding affinity of the immunoglobulins (spontaneously produced by B cells) for an exogenous antigen is low and insufficient to eradicate the pathogen carrying it. The chronic presence of the pathogenic antigen induces chronic transcription of Ig genes (hyper-transcription) and proliferation of B cells bearing low affinity antibodies, which are inadequate to counter the pathogen challenge/threat. Expansion of activated B cells reaches peripheral lymphoid organs where, promoted by antigen presenting cells, they encounter the biotic pollutant and undergo SHM and CSR processes. The mutations mediated by AID alter the specificity and affinity of the Ig protein for a “pathogenic pollutant” and generate a pool of B cell mutants (genetic plasticity). This model only partially explains the spectrum of somatic mutations observed in SHM during antigen-driven gene editing. In fact, the major mutations are represented not only by AID-mediated C-to-T (and correspondent guanosine-to-adenosine, G-to-A), but also by adenosine-to- guanosine (A-to-G) DNA mutations [96], pointing to the involvement of mutagenic mechanisms other than AID. In this regard, the adenosine deaminase enzymes acting on RNA (ADARs) have double stranded RNA binding domains and a C-terminal adenosine deaminase domain able to induce adenosine-to- inosine (A-to-I) RNA editing, finally read as A-to-G [9,10,96,97]. Recently Zheng and colleagues [97] have shown that ADARs can edit both the RNA and DNA moieties of RNA–DNA hybrids, raising the possibility that ADARs (as well as AID) driven by specific double stranded RNA transposon transcripts, could directly induce (A-to-I) DNA mutations. Alternatively, an indirect mechanism of DNA mutations involving a reverse transcriptase (error-prone DNA polymerase-η) and pre-mRNA template intermediates has also been suggested [9,10]. Regardless the molecular mechanism, analysis of the total mutation pattern indicates that the major transitions observed in SHM are A-to-G, C-to-T, and G-to-A, but not T-to-C [96], suggesting that ADAR-mediated A-to-G mutations may involve only one DNA strand, while AID-induced mutations (C-to-T) may involve both DNA strands (with a subsequent G-to-A mutations on the opposite DNA strand); a possibility that has already been suggested [10].

During immunoglobulin somatic hypermutation, several B cell clones with different affinity for pathogenic antigens are generated (genetic and phenotypic plasticity). Among the different B cells bearing different Ig mutants, only those carrying antibodies with a high affinity for the biotic pollutant proliferate and, finally, eradicate the pathogen. This ability enhances the organism’s chances of survival and has therefore been inherited, although it may fortuitously lead to the development of autoimmunity by generating autoimmune clones. This is an extraordinary mechanism of non-random, “on demand” mutation, in which environmental changes through a biotic “pollutant”, drive the cells to edit specific exons of a specific eukaryotic gene, leaving the rest of the B cell genome intact. The chronic presence of cell-interacting pollutants would induce an intronic retrotransposon-mediated gene/protein plasticity. Intriguingly, the main actor in these processes is AID, the ancestor member of the RNA-guided APOBEC family. SHM and CSR require complex interactions involving the selective recruitment of AID to transcriptionally active Ig regions. Subsequently, AID has been shown to translocate unidirectionally in concert with RNA polymerase during transcription of Ig genes and to catalyses cytidine deamination and C-to-T mutagenesis on the non-transcribed DNA strand of actively transcribed Ig genes [39]. This mechanism provides the biological plasticity necessary for environmental selection of phenotypes, ultimately yielding new inheritable and “environmentally independent” versions of Ig genes expressed on novel memory B cells; an example of “genetic assimilation” of acquired characteristics [25]. Finally, the mechanism is able to adapt the gene structure, in such a way that it generates antibodies that, “wrapping” around the antigen, are more functional in their response to new environmental challenges/stressors. How does this molecular mechanism target AID to Ig locus for CSR and SHM?

The molecular mechanisms that guide AID to repetitive switch (S) regions during CSR have been described by Zheng and colleagues [95]. CSR is a deletional-recombination process in repetitive DNA elements of S regions located upstream of each heavy-chain gene segment. AID deaminates cytosines within transcribed S regions, and the resulting point mutations activate the DNA double strand breaks required for CSR [95]. Importantly, the authors have demonstrated that RNA transcripts from intronic switch regions serve as molecular guides to target AID to S region DNA. Repetitive RNA transcripts derived from germline S intronic elements associate with AID and guide AID to the complementary S region DNA in a sequence-specific manner [95]. Moreover, the authors showed that DBR1, necessary for both debranching intronic lariats and retro-transposition [87,88] (see Box 4), was also required for AID localization to S region DNA during CSR [95], suggesting that stable intronic lariats, containing repetitive sequences of the S region, have to be processed by DBR1 before carrying out their targeting functions.

Taken together, these data indicate that the RNA-mediated mechanism of AID targeting to Ig S ssDNA regions is mediated by intronic repetitive (viral) sequences generated by Ig hyper-transcribed genes, i.e., inadequate genes which produce immunoglobulin proteins that are unable to respond to new environmental conditions (new pathogens). A similar mechanism of RNA-targeting can be hypothesised for other mutagenic functions of APOBEC family members, as suggested in the putative mechanism model described above. Nevertheless, differences in the RNA-binding sites among APOBEC/AID systems are expected. Indeed, based on time of APOBEC/AID and retrotransposon expansion (see above Section 3.5 and [36,41]), the difference between the long repetitive sequences, such as those in the S region of Ig locus, which possess specific binding sites for AID protein and the retrotransposon (sub)families with specific binding sites for the different APOBECs, is in fact justifiable [38,40,42,43,49,54].

Finally, it can be hypothesised that, thanks to intronic transposons, the majority of cell proteins may be modified in a similar manner as immunoglobulins, finally adapting their structures to a new “pollutant” interfering with their functions. In line with this hypothesis, one third of adaptive mutations affect virus-interacting human proteins, suggesting that they have occurred in response to viruses [98]. In this regard, two specific domains of transferrin receptor involved in virus binding have been shown to rapidly evolve in rodents; mutations at these binding sites were able to successfully block virus entry while preserving iron-uptake function of the receptor [99]. These adaptive mutations shaped by selective pressures exerted by viruses may therefore represent one of the most dominant drivers of evolutionary changes in mammals [98]. Hence, a mechanism developed to counter a biotic threat would be exapted and extended to abiotic pollutants for biological adaptive purposes. This hypothesis unveils a novel function of the “virus trained” immune system, a kind of “military” laboratory for the development of biomolecular technologies that are ultimately exapted for “civil” purposes. Finally, the new biological technologies developed through viral training, viral domestication and natural selection might be able to predispose life to counteract present challenges and to prevent future unpredictable biotic but also abiotic environmental threats.

## 5. Environmentally Induced Adaptation of Eukaryotic Inadequate/Hyper-Functional Genes by Retrotransposon-Guided Mutagenic Enzymes: What Might It Have Led To?

### 5.1. Retrotransposon-Guided Mutagenic Enzymes: An Ideal “Lamarckian” Tool of Evolvability and Plasticity. The Example of Polyethylene Metabolising Moth Larvae

As shown in the Figure 3, through a “boomerang” mechanism, the transposon-guided APOBECs may edit genes codifying for the protein involved in the initial binding with the pollutant without DNA duplication. Retrotransposon-guided APOBEC mutagenesis represents an inducible gene-targeting mechanism, which selectively edits hyper-transcribed genes, the ones that are inadequate to cope with emerging persistent and growing environmental stressors. Notably, these non-random genome editing mechanisms activated “on demand” in response to novel environmental pollutants are able to “choose” a few hyper-functional genes in which to create novelties, i.e., gene mutations (genetic plasticity). A recent evidence has revealed that different intronic transcripts from the same locus are tightly and differently regulated in mammalian cells [86]. Since retrotransposons transcribed from a specific intron would surgically direct a site specific mutagenesis in the intron and the adjacent exons, this makes it possible to modify specific protein domains. This means that mutations, originally occurring randomly (“Darwinianly”) during DNA duplication along the whole DNA molecule of viruses and prokaryotes, would surgically (“Lamarckianly”) take place in specific regions of the hyper-transcribed genes in the vast eukaryotic genomes (leaving intact the rest of the genome). The non-random genome editing mechanisms that I have described for humans would easily explain the incredible complexity of human protein structures and interactions. However, it is likely that similar mechanisms are present in other eukaryotic organisms, and altogether they might have provided a crucial contribution to complexity during “eukaryotic” evolution. Finally, the eukaryotic non-random genome editing mechanisms, providing high DNA evolvability and plasticity to the changeable environmental challenges on Earth, have likely allowed the development of the extraordinary complexity of higher organisms, which need large genomes to store a huge amount of information.

In line with this hypothesis, there is the new ability, developed by larvae of the wax moth, to metabolize polyethylene [100], a relatively recent man-made product. It is, in fact, possible that wax moth larvae in novel environmental conditions with increasing amounts of plastic derivatives (and the scarcity of wax) would have favoured polyethylene (non-specific) binding to some proteins of the wax metabolic pathway. It is known that high concentrations of molecules (e.g., polyethylene), whether they be pollutants or non-pollutants, induce low affinity (non-specific) bindings to cellular structures, such as those shown in Figure 3. Consequent gene hyper-transcription and protein synthesis upregulation would further favour the interaction with polyethylene. It is therefore possible that under conditions of steadily rising amounts of polyethylene, “non-specific” bindings could be at the origin of the induction of non-random genome editing responses. In the case of wax moth larvae, the plastic molecular interference may have pushed enzyme gene hyper-transcription, ultimately inducing the wax enzyme gene to modify into a gene encoding a polyethylene metabolising protein.

### 5.2. Non-random Genome Editing Mechanisms: Tools to Transform Pollutants into Essential Components of Life and Possible Links of Neotenic Traits to “Neotenic” Environments

Non-random genome editing mechanisms determine new protein structures and affinities for chronic pollutants. Structures that restore previous functional protein bindings in the presence of pollutants will have a selective advantage. Therefore, it is likely that protein structures that “accommodate” and make room for pollutants within cell structures, thus increasing cell protein contacts/affinities for pollutants, will be rewarded by Darwinian selection. Alternatively, also the loss of affinity, such as in the case of CCR5 alleles lacking HIV-1 recognition [13], may occasionally provide survival advantages to a cell/organism.

In the case of Figure 3B, as well as in Ig-SHM, gene editing mechanisms (combined with Darwinian selection) are able to increase protein affinity for the pollutant; the protein is shaped in a way that “accommodates” and integrates the pollutant inside it (similarly to how viruses are “accommodated” into a cell). In these cases, the mutated tissue becomes functionally “addicted” to the pollutant (without the pollutant the protein no longer functions) and strongly enhances its avidity for the pollutant, finally increasing pollutant tropism for the mutated tissue. This event leads to sequestration of pollutants (even at low concentrations) by the mutated tissue (e.g., from circulatory systems) and to a non-homogeneous distribution of pollutants. Notably, this occurs even for some essential exogenous (e.g., inorganic) components of cells. For example, iron is mostly stored in the liver, spleen, marrow and skeletal muscle as ferritin or haemosiderin. It is tempting to speculate that some essential inorganic components of organisms (e.g., O_2_, Fe, I) may have been toxic pollutants in the past and some current pollutants, by-products of anthropic “metabolism”, after opportune genome editing and selection will be essential components of future generations of organisms. The exaptation of toxic pollutants into essential components of life is similar to viral domestication, both ultimately produce an “environment internalisation” into biological structures.

However, to reach a specific tissue, exogenous biological components (or pollutants) have to be assimilated and transported to the tissue. Their tissue targeting can be mediated by a gradient passive diffusion after water solubilisation or suspension and the crossing of cellular lipid membrane barrier and/or by intermediate carriers with progressive binding affinities, which generates a sequestration gradient until the internal target tissue. This is evident, for example, in the haemoglobin-mediated oxygen transport to muscular myoglobin or in the case of transferrin-mediated iron transport to cellular iron-storage proteins (i.e., ferritin or haemosiderin) or to muscle myoglobin or to erythrocyte haemoglobins. Notably, in the case of deficiency of essential exogenous components (or dysfunction of intermediate carriers), their transport is impaired, leading to loss of function of the “addicted” proteins and tissues. This occurs, for example, in iron or iodine deficiency to haemoglobin and erythrocytes or thyroid hormones and thyrocytes, respectively. However, in the case of chronic deficiency, the tissue cells need to activate genome editing mechanisms in order to restore the function of the “addicted” proteins and tissues. In this case, the cells might be able to restore a “non-addicted” and “neotenic” form of the protein. Alternatively, the organism may be able to restore its functionality without the tissue whose survival depends on the exogenous component no longer available in the environment. In this regard, there are several examples of species that are unable to fully come into their adult form and retain juvenile characteristics (neoteny) because some nutrients are unavailable in the environment where they live. For example, in some amphibians, the larval neotenic trait has been associated with the aquatic environments of mountainous regions that are deficient in nutrients containing iodine. However, the larvae quickly begin metamorphosis and transform into terrestrial adults when iodine is administered [101], suggesting that their metamorphosis could be driven by the reactivation of the thyrocyte function, which is non-functional in high-altitude iodine-free environments.

It is tempting to speculate that “neotenic” forms of proteins, cells and organisms might derive from “neotenic” environments, i.e., environments deficient in a component that has become essential for a protein function and, consequently, for the survival of a tissue and the complete development of an organism. The neotenic trait would be a necessary delay in organism development due to deficiency in nutrient assimilation, and an advantageous and “selected” trait in species that move rapidly from one ecosystem to another, as do, for example, human beings.

### 5.3. The New Hypothesis of Giraffe Evolution and the Fascinating Hypothesis of “Selfish” Endogenous Retrovirus Reactivation by Starvation-Induced Gene Hyper-Transcription

When we consider deficiency of nutrients (a starvation stress conditions), the famous neck issue in giraffe evolution comes to mind. Interestingly, the increased neck length depends on an increase in vertebral dimensions and not on vertebral numbers. However, there is much more to explain than just the neck, as giraffe evolution involves its whole structure. In this regard, there is a pathological condition characterised by excessive growth of bones and muscles, known as gigantism, which is caused by over-production of growth hormone (GH) and down-stream hepatic production of fibroblast growth factors (FGFs). Interestingly, there is evidence of a correlation between prolonged fasting and a significant increase in GH and FGF21 levels in the blood of humans and mice [102,103]. The GH pathway is a regulator of organism dimensions and could be an optimal tool to adapt organism dimension to environmentally induced mass starvation. Indeed, when GH is upregulated, e.g., by fasting, the genes involved in the downstream pathway, either soluble factors (e.g., FGFs) or their receptors, are induced to (hyper)transcription. Among these genes, a species-specific “bottleneck gene”, whose epigenetic/homeostatic mechanisms of adaptation are the least flexible (“weak”), is the candidate for non-random gene editing transformation. Interestingly, through comparative animal genome analyses, several genes exhibiting mutations have been identified in the giraffe genome, including the *FGFRL1* gene, a member of the FGF receptor family. This receptor has a striking uniqueness in the domain that interacts with FGF ligands, making it a likely “bottleneck gene”, responsible for abnormal giraffe growth [104]. Therefore, hyper-transcription of the GH pathway genes and *FGFRL1* associated gene mutations could have played an important role in abnormal giraffe growth. Similarly, GH pathway might have been involved in the selection of Pygmy or Watusi phenotypes.

Notably, in starvation stress conditions, the death of some (inadequate) organisms of a species could be advantageous to species propagation. Indeed, when the dead/living being ratio is high (e.g., during epidemics and/or when populations have a small number of individuals) a significant increase in nutrient availability would occur, giving the species a better chance of survival. Under starvation stress conditions, a “selfish” ability to generate a virus-carrier organism that develops, contains and spreads new viral sequences (potentially able to kill its own kind except healthy carriers that spare themselves) acquires an evolutionary sense. In this scenario, I would not be surprised if in the future, it is discovered that under starvation stress conditions the cells of organisms are able to reactivate endogenous retroviruses and to generate new infective viruses. In line with this hypothesis, there is evidence that deprivation of essential amino acids (AAs) leads to the transcriptional upregulation of integrated viral genes, including ERVs [105,106]. This viral reactivation in response to essential AA deprivation is a highly conserved mechanism present in different mammalian cell types [106], and it is likely mediated by a general impairment of protein synthesis, possibly linked to hyper-transcription of intronic ERV. This “selfish” mechanism could have been positively selected because it is advantageous to species propagation in cyclically occurring starvation conditions. It could represent a last attempt of a species to survive.

### 5.4. Abundance of Ancient Pollutants/New Nutrients Could Lead to the Rise of “Clades” and Altruistic Biological Behaviours by Mean of Non-Random Genome and Epigenome Editing Mechanisms

The above observation of the potential ERV reactivation during starvation stress conditions raises the possibility that selfish and altruistic biological behaviour could be driven by different environmental conditions: scarcity/fast versus abundance/feast of nutrients/resources or other biological needs, respectively. I have already proposed that in virus–cell interplay, natural selection pushes them to symbiotic altruistic behaviours selected among myriads of unproductive struggles; however, to attain this altruistic behaviour, an initial abundance/expansion of a myriad of different virus and cell species is necessary. On the other hand, the expansion of biological species (microorganisms as well as organisms) can occur only when there is an abundance of (accumulated) nutrients required for their expansion. Such nutrients can reach the Earth from space (e.g., meteorites) or can be released by the Earth (e.g., volcanic eruptions) or can be continuously produced by biological structures (e.g., metabolic waste products). Notably, a geographical region that keeps their biological waste products by limiting their dispersion outside its border, tends to accumulate them and to transform an ecological niche (niche construction). However, the accumulation of substances in a specific ecological niche can occur when those substances are also chemically stable and non-metabolisable by organisms (i.e., non-transformable), such as polyethylene nowadays (or nucleotides and amino acids four billion years ago, see later Section 8.6). However, the appearance of new biological species (from outside the niche or evolved from organisms in the same niche) able to exploit the accumulated waste products (pollutants) for proliferation purposes will produce the expansion of the new species (eutrophication). I have already suggested how the presence of a high concentration of pollutants favours “non-specific” low-affinity bindings that, interfering with biological structures, induce non-random genome editing mechanisms to transform genes and proteins interacting with pollutants. Along this process, the accumulated waste products (pollutants) become substrates; the larvae of the wax moth that develop the ability to metabolize polyethylene [100] could be an example of this. However, when new biological species acquire the ability to enzymatically metabolize accumulated substrates, two concomitant processes could be underway. On one hand, the new species may gain energy from substrate metabolisation and rapidly proliferate and, on the other hand, they start to chronically (hyper)transcribe the genes involved in the new metabolisation pathway. As already described, gene hyper-transcription can induce non-random gene editing mechanisms to generate different gene mutations and different “clones” of cell species (similarly to somatic hypermutation of immunoglobulin genes). Finally, the two processes can induce the proliferation of biological species with different versions of genes along the metabolisation pathway; a “clade”, to borrow a word from Elderidge–Gould. Therefore, during this expansion phase, different genetic attempts of the same viral, cellular or organismal “family” (relatives) would be expected to expand. In this case, environmental pressure pushes a single species to generate several different adaptive attempts (cladogenesis) rather than one single species (anagenesis). However, if the new substrate is limited, the expansion of the new species progressively reduces the amount of the substrate, the expansion phase slows down and, resembling the immune system response to pathogens [94], leaves room for competition and contraction phases. During these phases, the groups of biological relatives will compete and fight for the same accumulated substrate. Among the different family versions, only those that more efficiently adapt to the new environmental condition would survive (natural selection) long enough to leave fossil records of themselves. Notably, during the process of contraction some species may survive exploiting the “work” performed by some new specialised species. The first cell/organism able metabolize a waste product (an initial incidental event) determines an environmental change (niche construction) that influences the neighbouring cells/organisms that directly or indirectly interact with the new species. Indeed, the appearance of a new species able to efficiently metabolize the new substrate (“ancient” waste product/pollutant) into an old substrate gives new opportunities of survival to some “relatives” unable to metabolize the new substrate. A phenomenon that has been well described in microorganisms (e.g., the “cheater” mutants of bacterial strains [107]). Therefore, among the different surviving species of the same “family”, it is possible that an “altruistic” intra-species behaviour (“pre-symbiosis”) might develop under “selfish” natural selection. However, altruistic intra-species behaviours can occur either among related microorganisms with differences in a gene (e.g., bacterium clones [107]) or among genetically similar (or identical) eukaryotic cells and organisms (e.g., tissues in an organism and social insects/animals). Indeed, the different tissues altruistically cooperate to make the extraordinary “phenomenon” of organismal life possible. This altruistic cooperation among tissues that share the same genomic background in an organism (e.g., the cooperative behaviour of immune system in response to pathogens) is basically mediated by epigenetic signatures that drive tissue differentiation. However, significant differences in epigenetic signatures are responsible not only of different phenotypes/behaviours of tissues/organs but also of distinct caste in social insects. Indeed, the different caste phenotypes (as “migrating” organs), which come from the same genotype, are, at least in part, determined by differences in DNA methylation [108]. Again, an initial incidental event (a new-born cell or insect), which determines an environmental change, may influence the epigenetic signatures of subsequent new-borns (new neighbouring cells/organisms) that directly or indirectly interact with the earlier new-born cells or organisms. To perform an altruistic cooperation, the epigenetic signatures must be well organised according to a “rule” stored in an “epigenetic memory”. In this regard, it is possible to imagine that the huge amount of information accumulated and stored in eukaryotic genome/epigenome has allowed the development of a collaborative and altruistic behaviour, an intra-species symbiosis. This would represent an alternative altruistic process to classical inter-species symbiosis that is mediated by epigenetic regulation (e.g., promoter/enhancer methylation/de-methylation). Taken together, these observations may provide the rationale for Hamilton’s rule and sociobiological evidence of the evolution of altruism and cooperation (from microorganisms to organisms) among genetically related species [107].

Notably, epigenetic differences are also responsible for secondary sexual traits and gender diversity. Therefore, epigenetic driven gender diversity may have also been selected for its propensity to generate an (intra-species) altruistic cooperation, as for classical symbiosis (e.g., virus–cell symbiosis is a sort of primordial form of sexuality). Indeed, the whole human population with its specialisation is an excellent example of intra-species altruistic cooperation; human beings all together constitute an efficient and collaborative organisation of specialised “tissues” that is based on different cognitive skills. Finally, four main levels of collaborative altruistic behaviour could be defined: (1) inter-genomes (inter-species collaboration among different genomes or symbiosis); (2) inter-epigenomes (intra-genome collaboration among different epigenomes, tissues); (3) inter-genders (intra-species collaboration between different genders); (4) inter-skills (intra-species collaboration among individuals with different cognitive skills).

It is, however, hard to explain how the complex organisations based on gender diversity as well as on distinct tissues in an organism or on distinct castes in social insects were developed. Nevertheless, drawing a comparison with non-random genome editing mechanisms, it is possible to hypothesise the involvement of non-random epigenome editing mechanisms, which could lead to the generation of “clades” of epigenetic attempts that were subsequently selected and stored in an “epigenetic memory”. In Section 6.2, I will describe how non-random genome editing of regulatory sequences, transcription factors and transposon transposition might mediate the “memorisation” of epigenetic changes, thus accounting for the epigenetic inheritance. Moreover, the concept of epigenetic memory [109] will be discussed later in the Section 7.2. Now, some negative aspects stemming from non-random genome editing mechanisms are instead introduced.

### 5.5. Chronic Diseases as by-Products of Environmentally-Induced Non-Random Genome Editing Mechanisms in Environmentally Inadequate Genomes

I would now like to make some further observations that will help us to fully grasp some other consequences of the retrotransposon-guided mutagenic enzymes. The example described in Figure 3 depicts a general signalling protein involved in pollutant binding; however, depending on the stereochemistry of both the pollutant and polymorphic protein domains (dependent on the genomic background), virtually all types of proteins with different functions may be able to bind to pollutants (i.e., enzymes, transcription factors, receptors, hormones, onco-related proteins etc). Importantly, through non-random genome editing mechanisms, pollutants that do not directly bind to DNA, would be potentially mutagenic and able to induce DNA mutations in both alleles of hyper-transcribed genes present in tissues interacting with the pollutant. The chronic interaction between a pollutant and an exon-derived protein domain would be the trigger for DNA editing induction; therefore, the tri-dimensional structures and arrangement of specific atoms in the space (i.e., their stereochemistry) of both tissue proteins (whose structures mainly depend on exon gene polymorphisms) and pollutants, would be the initial trigger of non-random DNA editing mechanisms. Notably, after mechanism induction, the intronic transposon polymorphisms can be crucial to guiding, or not guiding, APOBEC family members or other DNA mutagenic enzymes (for example ADARs or LINE-1-encoded ORF2p) to target sites, ultimately generating biodiverse responses and different inter-individual gene-editing attempts. Among the different mutants, “aborted” proteins and proteins with different affinities for the pollutant would be generated and cell bearing mutated proteins that enhance cell fitness and cell survival in the presence of the pollutant (new cell environment) would be selected and expanded, generating a mutated tissue (a first level of selection). Although mutations in tissue-specific genes will be expressed only in specific tissue cells (mutated tissue), a tissue phenotypic variation may affect neighbouring/interacting cells of other tissues in an organ, finally involving the whole organism. Indeed, within the organ, the mutated tissue interacts with neighbouring cells of other tissues, only those restoring organ function in the presence of the pollutant (new organ environment) would allow organism survival, while in some cases might lead to organ and organism failure (a second level of selection). Finally, among the different inter-individual tissue/organ attempts, only those producing lasting fitness of the organ and thus of the organism in the new “polluted” ecosystem would be Darwinianly “inherited” (or “Waddingtonly” genetically assimilated). It is, in fact, likely that the most favourable mutations for the organisms would then survive long enough to be passed on, whereas mutations that are unfavourable or less favourable to the organisms (organisms bearing environmentally inadequate genomes) may produce rapid death or diseases (a third level of selection). The mechanisms through which some new somatic genes as well as new regulatory sequences might be inherited by the progeny will be discussed in a subsequent chapter (Section 7).

As already indicated, during biological adaptation to new environmental conditions many aborted attempts and organ and organism failure can be generated, raising that possibility that in some cases adaptive mechanisms could lead, not only to the organism death but also to pathological conditions. Indeed, transposons and mutagenic enzymes have been shown to be involved not only in species adaptation and evolution, but also in chronic diseases. Can non-random genome editing mechanisms unveil a link between pollution and chronic diseases? In support to this possibility, there is epidemiological evidence of an association between air pollution exposure and the risk of developing Alzheimer’s disease that suggests an environmental involvement in the disease development [110,111]; but even more striking is a recent investigation showing that in the pathological neurons, the Alzheimer’s disease-related *APP* (amyloid precursor protein) gene occurred “mosaically” as thousands of genomic variants [112]. Notably, the *APP* gene mutants were promoted by neuronal *APP* gene (hyper)transcription, suggesting that they might represent the result of chronic attempts of adaptation (possibly promoted by non-random genome editing mechanisms) to pollutants interfering with the APP protein. Of note, some genetic attempts could generate new proteins and/or pollutants bound to new proteins (i.e., new haptens) recognised as extraneous (non-self) by the immune system, ultimately leading to autoimmunity. Indeed, concomitant activation of inflammatory immune pathways is a common feature in individuals with chronic diseases. It is tempting to speculate that in these cases, the immune system might function as a scavenger by eliminating evolutionarily/environmentally “inadequate” and “unwanted” attempts. If this is the case, the immune system would finally allow to select the most self-compatible elements rather than to discriminate between self and non-self biological structures (indeed, in this last case, symbiosis would be unexplainable).

Altogether these observations suggest that an organism will survive the challenges posed by the environment (e.g., a new pollutant or a new virus) only in the case in which its genome intrinsically possesses the ability to adapt to. On the contrary, if the genome of an organism does not possess such an ability, the organism (owning it) is inadequate for the environmental challenges and it will finally be eliminated with its genome. From this point of view, chronic diseases such as cancer may not be related to dysregulation of non-random genome editing mechanisms (e.g., non-Ig off-target mutagenic responses), as previously suggested [10] rather they may represent inadequate responses to the environmental challenges generated by cells bearing an evolutionary inadequate genome, i.e., unable to solve the “problems” imposed by the new stable environmental conditions. Therefore, within the considerable and growing heterogeneity of organisms, there are evolutionarily adequate genomes, i.e., able to solve the unpredictable environmental conditions, and evolutionarily inadequate genomes that, leading to gene/protein/cell/tissue/organ/organism failure, will be finally eliminated. The possibility that non-random genome editing mechanisms could lead to chronic diseases warrant further investigation and will be addressed in future perspective paper; the present work is instead aimed to investigate other evolutionary aspects. In this regard, cancer cells, which represent cells chronically “stressed” by organism environment (e.g., by immune system), will be exploited as model of eukaryotic cell evolution in the following chapter.

## 6. Exploiting Cancer as a Model to Study Non-Random Genome Editing Mechanisms and Eukaryotic Cell Evolution

### 6.1. A Cell Species That Exploits Non-Random Genome Editing Mechanisms to Survive in Chronic Stress Conditions: Cancer

Every “outside-in” signalling event from the environment to the cell yields an “inside-out” response from the cell to the environment and vice versa, leading to a fluctuating “epigenetic” balance or a search for a new “genetic” balance. Indeed, both epigenetic and genetic responses can be sequentially induced by environmental changes. An initial cause produces an effect which, through a boomerang reaction, influences the starting cause, creating negative or positive feedback loops that sometimes make it difficult to determine what the original cause (if there is one) and the cause-effect relationship of a phenomenon are. Epigenetic mechanisms are induced first, they are usually reversible and tend to reduce external changes around a fluctuating balance through “reactionary” negative feedback loops. Conversely, when a gene mutation is incidentally induced, for example, by a viral infection (environment), the mutation indirectly produces an environmental change (environmental plasticity), a process whereby a mutated cell actively alters its environment, a sort of cellular niche construction, that gives rise to “revolutionary” positive feedback mechanisms. Such positive feedback mechanisms can be readily observed in metabolic diseases, for example, the “environmental” toxicity of phenylalanine accumulation in phenylketonuria is a consequence of an enzymatic mutation and loss of function; however, this same phenomenon occurs in other biological contexts, including cancer. When a normal cell is transformed into a cancer cell, the environment suddenly becomes hostile, as immune cells and eventually drugs start to “threaten” cancer cell expansion [113]. In some ways, cancers have a lot of functional similarities to viruses. Both can kill their host, produce genome selections, spread their deadly kiss and are challenged by immune system. Indeed, it has been suggested that cancer extracellular vesicle (exosomes) spreading could be involved in the process of metastasis [114]. Moreover, it has been proposed that oncogenic nucleic acid materials and mutagenic enzymes contained in extracellular vesicles (forms of eukaryotic horizontal genetic transmission) spread the tumour and its deadly activity to other tissues [37,114], further highlighting virus-cancer similarities. In solid tumours, the disorganised three-dimensional structure creates cancer hypoxia and nutrient starvation, stress conditions that further drive cancer cell adaptation. However, to adapt to chronic and adverse environmental conditions, cancer cells likely activate the same smart non-random genome editing mechanisms, already described in normal cells, which promote cancer genome heterogeneity and ultimately cancer cell adaptation. There is in fact evidence that cancer cells use non-random DNA editing mechanisms, producing non-random clustered mutations similar to those generated in highly polymorphic human genes, such as those of *HLA* and *KIR3DL1/KIR3DS1* [115].

It is well known that cancer cells can exploit normally dormant embryonic pathways, acquiring embryonic and neotenic features (i.e., delaying maturity and retaining immature features) that foster developmental plasticity and face stressful environments. Indeed, cancer cells could represent new aggressive tissue species that rapidly evolve in a hostile “ecosystem”, the organism. Although at a different level of organisation, cancer cells behave similarly to an aggressive virus or a predator (like humans) that transforms or kills its “ecosystem”, the cell or ecological niche, respectively. From this standpoint, it is not surprising that under selection pressure, gene copy numbers of cancer cells diverge in subpopulations distributable in phylogenetic trees similar to those described for higher organisms [116]. The pattern of cancer evolution is characterised by the sudden emergence of new tumour clones, a pattern defined as a ‘punctuated clonal evolution’ [116] borrowing the concept from the non-gradual species evolution observed in fossil records [19]. Moreover, saltational evolution (a sudden multimutation leap) has been suggested to play a role in stress-induced mutagenesis of tumour cells [7]. Indeed, tumour mutation rate, possibly due to the stress conditions above described, is substantially elevated compared to that of normal tissues and this may account for the increased probability of multimutational leaps in cancer cells [7]. Hence, cancer appears to be a good model to infer eukaryotic cell evolution.

From this point of view, it is therefore possible to verify the behaviour of previously hypothesised mechanisms of non-random genome editing using cancer as a model for eukaryotic cell evolution. In this regard, the development of drug resistant cell lines is a good example of cancer adaptation and the mechanism of *P53* non-random gene mutation induced by doxorubicin in the MCF-7 breast cancer cell line is currently under investigation. Chronic administration of increasing doses of doxorubicin induces doxorubicin-resistant MCF-7 cell lines with a reproducible identical C to T point mutation at the end (the last nucleotide) of intron 4 of the *P53* gene. The specific intron 4 mutation of *P53* results in an abnormal splicing transcript with a 21-nucleotide deletion and in p53 protein loss (or gain) of function [117,118]. The mutation signature mediated by a consensus sequence of 4 (GTCA) nucleotides suggests the involvement of APOBEC3B [119], a well-known mutagenic enzyme associated with breast cancer and upregulated by doxorubicin [120]. Chronic DNA damage induced by doxorubicin likely upregulates the genome guardian *P53* exon and intron (hyper)transcription. It is therefore possible that the (hyper)transcribed *P53* retrotransposons, in particular the Alu element (with its unique sequence) present in the intron 4, could bind and surgically drive APOBEC3B just upstream of the mutation site of the *P53* gene, ultimately inducing the specific non-random point mutation and the alteration of a specific p53 protein domain.

### 6.2. Non-Random Retro-Transposition of Repetitive Elements and Non-Random Genome Editing of Regulatory Sequences and Transcription Factors Account for Non-Random Novel Regulatory Gene Networks and Epigenome Editing/Remodelling and Enheritance: The Example of Melanism in the British Peppered Moth

Figure 3B shows how the constant presence of a protein-binding pollutant interaction could potentially induce hyper-transcription and mutation of both copies of alleles encoding the protein without cell proliferation. However, under deep environmental changes (due, for example, to heavy industrial pollution), it is likely that several genes are induced to hyper-transcription by different pollutants. Therefore, several intronic transposons from hyper-transcribed genes could potentially generate multiple mutations (in one genome without cell proliferation), accounting for saltational evolution. Since the chromatin of hyper-transcribed genes is in an open state and accessible even for transposition, a preferential exchange of retrotransposon “regulatory sequences” among the hyper-transcribed genes would likely occur. Retro-transposition among hyper-transcribed genes would generate new introns and/or new response elements in promoter and/or enhancer regions not only of genes, but also of retro-pseudogenes, possibly generating paralogous genes and fusion proteins (e.g., from hyper-transcription of multiple subunits of protein complexes). Notably, this non-random retrotransposon exchange could produce novel regulatory gene networks, where a single transcription factor, originally associated with a specific gene, may link previously unrelated hyper-transcribed genes through a common response element (similarly to a poly-cistronic gene), ultimately reshaping epigenome pathways (non-random epigenome editing mechanisms). In addition, non-random retro-transposition exchange may potentially involve both paternal and maternal homologous chromosomes bearing hyper-transcribed genes, equipping the genes of both homologous chromosomes with the same regulatory response elements (introns, promoters, enhancers). A phenomenon that might precede chromosomal translocation homozygosity events (see subsequent Section 6.3). This new network mediated by a common transcription factor would be functional in accelerating and coordinating the cell response to a new specific environmental pressure produced by several different pollutants, and therefore this mechanism might have been positively selected during evolution. Notably, these novel regulatory networks, although independent of gene mutations, would be “irreversibly memorised” in non-coding regions of the genome and therefore could generate inheritable and persistent (“genetically assimilated”, to borrow a word from Waddington) alterations in the control of gene expression (an epigenome remodelling). These memorised changes in regulatory networks might be inherited by progeny, accounting for the well-known “epigenetic” transgenerational inheritance [22,121]. In this regard, it is noteworthy that the mutational event responsible for the melanism in the British peppered moth during industrial environmental changes is the insertion of a transposable element [21], raising the possibility that environmental changes may have led to the generation of a novel regulatory gene network mediated by this transposon transposition. Notably, a pollutant-driven hypothesis of peppered moth melanism, as a selective mechanism in addition to differential bird predation by crypsis, has been proposed by Riley [122]. Since melanin has been shown to be able to chelate metal ions, an increase in melanisation has been suggested to produce a localised accumulation of metals and potential cytoprotective effects in moths exposed to toxic levels of heavy metals [122]. In addition, it can be hypothesised that the non-random genome editing mechanisms induced by pollution could have selected both novel response elements for melanin gene transcription directly dependent on heavy metal presence and mutated melanin forms able to more strongly sequester heavy metals. The consequent addiction to heavy metals of the mutated melanin transcription and therefore of the peppered colour of the moth would be consistent with the recent and rapid reversal (“neoteny”) of the process (the decline in the frequency of peppered forms) associated with a reduction in atmospheric pollution [123].

I have already suggested that viral repetitive sequences inserted during viral infection or retro-transposition into intronic regions of a gene are expected to be similar to those inserted in promoter and enhancer regions of the same or other simultaneously hyper-transcribed genes. Hence, such promoters and enhancers might also be targets of mutagenic enzymes driven by intronic retrotransposons of hyper-transcribed genes. In this regard, members of the AID/APOBEC family have been shown either to efficiently deaminate methylated cytosines, leading to methyl-cytosine demethylation [124], or to induce C-to-T somatic mutation in non-coding promoter regions, generating novel binding sites for (oncogenic) transcription factors [125]. Notably, the ability of AID/APOBEC family members to de-methylate methyl-cytosine in regulatory (promoter or enhancer) regions might promote gene transcription. In this regard, it has been shown that APOBEC3B is able to increase expression of oestrogen-responsive genes in a model of breast cancer, [126], suggesting that APOBEC3B might actually de-methylate oestrogen-responsive regulatory elements in breast cancer cells. The possibility of de-methylating methyl-cytosine and editing C-to-T in the CG-rich islands of promoters and/or enhancers can promote respectively either epigenetic and reversible (“environmentally dependent”) increased gene transcription or (similarly to retro-transposition) generation of “irreversibly memorised” (or “genetically assimilated”) novel responsive elements and regulatory networks (new epigenome pathways). These AID/APOBEC activities in the regulatory regions of the genome point to additional roles for AID/APOBECs (including transcriptional regulation) that, in these cases, work as effectors of epigenome regulation and remodelling. Notably, the generation of new repetitive viral sequence in the regulatory regions can be inherited by cell progeny and may account for the “epigenetic” inheritance. On the other hand, during chronic stress, *APOBEC* genes themselves as well as genes of transcription factors (both effectors of epigenetic regulation) are hyper-transcribed and therefore these same genes and even their regulatory regions can be targets of retrotransposon-mediated mutagenesis and/or of retro-transposition of new response elements, potentially leading to new APOBEC and transcription factor members and new regulatory gene networks (new epigenome pathways). The chimeric *APOBEC3A-3B* deletion variant as well as p53 transcription factor mutations, which are linked to a higher risk of developing cancers, could be examples of these phenomena [118,127]. Notably, the reciprocal potentiation of mutagenic enzymes and endogenous retroviral sequences in eukaryotes allows the generation of new genes and responsive viral elements with new unique sequences without viral infection (viruses might not be necessary anymore), making eukaryotic life stronger and stronger until the combinatorial possibilities of a finite DNA content have been exhausted (if this will ever occur). However, C-to-T somatic mutations in non-coding regulatory regions [125] and new *APOBEC* and transcription factor gene variants [127] have been shown on cancer cells, raising the question of whether these phenomena are limited to pathological conditions or could be common aspects of eukaryotic cell development/evolution.

### 6.3. Non-Random Chromosomal Rearrangements and the Involvement of Non-Random Epigenome Editing Mechanisms in Cancer Cells Following Exposure to Chemical and Physical Stressors

I have already suggested how gene hyper-transcription and transposon transposition among hyper-transcribed genes would disseminate common response elements into promoter, enhancer and intronic regions of hyper-transcribed genes. However, what could be the mechanism leading to chromosomal translocation?

Gene hyper-transcription can be induced by chronic stimulation of transcription factors, and it is well known that transcription factors also induce chromatin remodelling [128], a step which precedes chromosomal translocations [77,129]. Using a model of prostate cell cancer, it has been shown that the androgen receptor (AR) activated by dihydrotestosterone (DHT) induces both specific gene transcriptions (working as a transcription factor) and chromosomal movements that create either mono-allelic or even bi-allelic spatial proximity among genes bearing the same intronic androgen response elements (AREs) [77]. Notably, ARE repetitive sequences are one of the hormone response elements typically located within Alu repeats [59,130]. In this model, upon DHT stimulation, AREs of different genes appear to move towards a common chromatin region by mean of a nuclear myosin/actin-dependent mechanism [77], indicating that AR transcription factors bound to AREs are able to move AREs of different genes to a chromatin area in which DHT-AR-ARE(Alu) complexes are accumulated. A mechanism that calls to mind the chromosome movements in the mitotic spindle. Notably, the concomitant stimulation of DHT with genotoxic stressors produces both the proximity of ARE from distant chromosome regions and the recruitment of genotoxic stress-induced enzymes, AID and LINE-1-encoded ORF2 endonuclease [77]. This association facilitates the generation of DNA double-stranded breaks and subsequent specific non-random translocations (even on both homologous chromosomes) mediated by the non-homologous end joining (NHEJ) machinery [77]. A mechanism that provides the rationale for Alu repeat enrichment in fragile chromosomal sites, DNA regions with increased loss-of-heterozygosity frequency [131,132]. Notably, the observed gene fusion events have been found to be present in 50–70% of prostate cancers in vivo [77], indicating that this model of tumour translocations mimics the in vivo events.

Interestingly, both chemical (Etoposide and Doxorubicin) and physical (50Gy of ionising radiation) genotoxic stresses have been shown to reproducibly produce the same chromosomal translocations when combined with DHT [77]. This is a surprising event because high doses of ionising radiation (50Gy, produced by stochastic radioactive decay) do not produce random DNA damage as expected, but rather the same reproducible translocations (unique non-random DNA damages), suggesting that eukaryotic as well as prokaryotic cells may perish or may correctly repair and manage most random DNA damage produced by ionising radiation! This surprising aspect is discussed in Box 6.

Box 6 **Inhibition of mutagenic enzymes as a possible tool to prevent genetic effects of ionising radiation in eukaryotic organisms.**

Life was threatened in its early stages by direct and indirect ionising radiation damages and likely developed and memorised mechanisms to control it early on. In this regard, the surprising tolerance to high dose ionising radiation of preimplantation stages of embryonic development in rats and mice suggest that these early eukaryotic stages of development have the ability to “manage” ionising radiation damage, an ability that may be lost in later stages. It was thought that during the preimplantation period some pluripotent stem cells could repair radiation damage and compensate for the death of other embryonic cells until a threshold limit, which determines either embryonic death or embryo survival without any detectable malformations, for which Russell coined the “all-or-none-rule” i.e., “killing or normality” [133]. Since radiation per se does not seem to be able to induce random DNA damage, the non-random DNA damages (i.e., chromosomal rearrangements) are likely an indirect by-product response to a general (chemical or physical) genotoxic insult possibly mediated by non-random genome editing mechanisms (i.e., by transposon-driven AID/APOBECs, ADARs or ORF2p). I have already described the mechanism of non-random genome editing activation when is induced by chronic presence of high concentrations of molecular pollutants (e.g., chemical drugs, see Figure 3); however, similar considerations can be made with regard to administration of chronic high dose of “physical pollutants”, such as ionising radiation or electromagnetic waves from cell phones. This possibility suggests that drugs able to inhibit mutagenic enzymes might be utilised not only to inhibit cancer development [5,134], but also to inhibit mutagenesis in elevated radiation (or chemical drug/pollutant) risk conditions, such as in radioactive contaminated areas or on a journey to “colonize” other planets.

To generate non-random chromosomal translocations, sequential events that induce proximity of intronic response elements (e.g., ARE) and recruitment of genotoxic stress-induced mutagenic enzymes have been shown [77]. In a prostate cancer model, the recruitment of LINE-1 ORF2 protein and AID mutagenic enzymes have been described to act through two independent mechanisms [77]. Therefore, the recruitment of AID, as in the case of CSR, might be mediated by its binding with intronic repetitive transcripts, while ORF2p may be mediated by its binding with intronic LINE-1 transcripts, both transcripts possibly originating from hyper-transcribed genes. Alternatively, AID and ORF2p might be independently driven to specific DNA regions through a direct binding with AR, as hypothesised for APOBEC3B and the oestrogen receptor in a breast cancer model [126]. However, in the case of prostate cancer cells, AR should have specific binding sites for both AID and ORF2p (beside AREs) [77]. Lastly, an indirect binding mediated by different RNA repetitive “bridges” of AR with AID and/or ORF2p can be also hypothesised. I have already suggested that transcription factors and anti-viral APOBEC members might evolve from two distinct viral nucleoproteins, both with an intrinsic ability to bind to repetitive nucleic acid sequences. Therefore, some viral sequences inserted into intronic regions might have been positively selected and “domesticated/educated” during evolution for their intrinsic functional ability to bind to both nucleoprotein-derived transcription factors and nucleoprotein-derived anti-viral enzymes, such as the mutagenic members of AID/APOBEC (or ADAR) family or the ancient (ORF2) endonucleases. These types of intronic repetitive sequences would be able to create bridges between different RNA binding proteins (e.g., transcription factors) and stress-induced mutagenic enzymes, depending on the intronic transposons of (hyper)transcribed genes. In this regard, it has been shown that AID translocates unidirectionally along with RNA polymerase during Ig gene transcription, but it does not interact directly with RNA polymerase [39], suggesting that a specific (intronic) RNA repetitive sequence may mediate the interaction between AID and RNA polymerase during transcription. From this point of view, the intronic RNA transposons would represent versatile modules, which, varying not only among different genes of the same individual but also among different individuals in the same gene, would finally lead to different individual responses to the same stress condition.

Regardless of the way that the genotoxic stress-induced enzymes are recruited, non-random chromosomal translocations appear to be induced by two independent mechanisms. On the one hand, the genotoxic stressors induce mutagenic enzyme expression and on the other hand, the transcription factors (in this case DHT-AR) induce specific gene hyper-transcription, transposon expression and transposition among hyper-transcribed genes. The target specificity of non-random chromosomal translocation would be assured by the presence of common response elements (e.g., ARE/Alu elements with a unique sequence), previously disseminated by non-random retro-transposition of repetitive elements into promoter, enhancer and intronic regions among the hyper-transcribed genes (see Section 6.2). These types of events can produce similar translocations on both paternal and maternal homologous chromosomes, a sort of “cell speciation” occurring without cell proliferative selection (cell cycle–independent) in a single-hit process.

### 6.4. Solid Cancer/Leukaemia/Lymphoma as Models of “Cell Speciation” and the Example of Mouse Speciation in Seveso

Chromosomal translocations are usually pathogenic rearrangements often involved in cancer and, in particular, in leukaemia and lymphoma development; however, they are also present in healthy individuals and in cord blood samples from healthy new-borns [135], suggesting that translocations are not themselves sufficient for the transformation of a normal cell into a malignant cell, and pointing to the involvement of the cell environmental context, i.e., the organism in which the chromosomally rearranged cell lives. Indeed, there are several examples of non-pathogenic chromosomal imbalances with no phenotypic effects that are directly transmitted from parents to offspring [136]. In this regard, Robertsonian translocations (fusions between two acrocentric chromosomes), which can potentially lead to speciation [20], are the most common chromosomal rearrangements in humans, occurring in approximately one in every thousand human new-borns [80]. When the Robertsonian translocation is balanced, the person carrying it (carrier) has a full genetic complement and is healthy. Nevertheless, Robertsonian translocation carriers usually have a reduced productive fitness due to meiotic segregation disturbances and inter-chromosomal effects, leading to a higher probability of genetically imbalanced gametes [80]. Indeed, sperm of Robertsonian translocation heterozygotes have a proportion of unbalanced chromosomal complements, leading to the increased risk of a chromosomally unbalanced foetus, infertility and miscarriage [80]; however, all sperm of Robertsonian translocation homozygotes are balanced, pointing to Robertsonian translocation homozygosity as a potential mechanism of human cell speciation [80]. Interestingly, homozygosity for a particular Robertsonian translocation chromosome, which theoretically can lead to the establishment of a new species, is unlikely to occur through an intermediate heterozygous translocation stage because this stage is self-sterilising. Indeed, heterozygote infertility generates a reproductive isolation mechanism known as hybrid incompatibility, which suggests the possibility that balanced translocations of both homologous chromosomes may occur in a single-hit process. In this regard, I have previously hypothesised that both paternal and maternal homologous chromosomes bearing hyper-transcribed genes can non-randomly exchange retrotransposons, thus equipping genes of both homologous chromosomes with the same regulatory response elements (introns, promoters, enhancers), which may ultimately mediate the direct generation of homozygous translocations in the absence of (sterile) heterozygous intermediates.

Finally, chromosomal translocations produced by non-random genome editing mechanisms would generate new persistent junctions that would reduce the distance among the genes bearing the same response elements, further accelerating the cell response to environmentally induced transcription factors. In the case in which translocations occur in a single-hit process in both paternal and maternal homologous chromosomes, rearrangement homozygosity could be a potential mechanism of “cell speciation”. Indeed, when the chromosomal translocations produce a new “species” of tissue cell that is more suitable to survive in the new organism environment, it substitutes the old tissue species. However, the new cell species can be either incompatible with the organism survival, as in the case of cancer/leukaemia/lymphoma (cell-mediated diseases, some of which may be environmentally induced through non-random genome editing mechanisms), or compatible and possibly advantageous to the organism, as likely occurred in the chromosomal speciation observed in mice after the Seveso disaster [20]. In the latter case, it can be hypothesised that non-random genome editing mechanisms may have produced mice both with dioxin-induced chromosomal translocations and with higher resistance to dioxin toxicity.

The chromosomal speciation that occurred in mice in Seveso is a clear case of inheritance deviating from Mendelian laws that occurred in wild house animals chronically exposed (and not protected as humans) to dioxin. However, non-Mendelian inheritance must be transmitted through germline cells; therefore, it can be a consequence of events occurring directly in germline cells or indirectly transferred from somatic to germline cells, which implies a somatic-to-germline transmission of genetic information. To generate a new species both paternal and maternal chromosomes have to be rearranged; however, the feature of hybrid incompatibility, for which heterozygotes are usually sterile or inviable intermediates, suggests that both paternal and maternal chromosome rearrangements must be induced in a single-hit process, as in the case of the cancer associated chromosomal translocations mentioned above [77]. Translocations causing hybrid incompatibility drive populations to maintain their genetic divergences, finally promoting speciation by acting as a reproductive isolating barrier. Notably, in the case in which a newly evolved species is more suitable to a new environment, hybrid incompatibility promotes the expansion of the most suitable species, by blocking the generation of the hybrid progeny that carries an inadequate genomic complement in one of the two chromosomes. A feature that accelerates evolution of the species and their adaptation to changing environmental conditions; this is likely the reason for positive selection of hybrid incompatibility.

## 7. Sexual Symbiosis: An Altruistic Cooperation of Distinct Sexual Functions in Order to Transfer Environmentally Induced Acquired Novelties to Progeny

### 7.1. Non-Mendelian Transgenerational Inheritance Induced by Chronic Environmental Changes: The Central Role of Male Germline Cells

How might non-Mendelian transgenerational inheritance occur? Notably, unlike female germline, male germline supports continuous generation of germline cells in adults. Therefore, differently from female germline cells, the male ones can sense environmental changes “in real-time” and are potentially sensitive to pollutant-mediated genome editing mechanisms, which have been suggested to be the main generators of biological novelties. Indeed, the preferential transmission of biological novelties from somatic cells down to male germline cells have been already suggested [9,137,138]. In this regard, spermatozoa from virtually all animal species have been shown to have a particular feature, i.e., the ability to spontaneously take up somatic extrachromosomal nucleic acid information present in extracellular vesicles, which may be delivered to fertilised oocytes and embryos [137,138]. However, like viral infection, the tropism of extracellular vesicles to a specific cell target depend on surface molecules (receptors/ligands) able to bind to specific ligands/receptors on sperm cell surface. In this regard, during spermatogenesis, spermatogonia undergo dynamic DNA methylation and chromatin reorganisation, which are associated with stage-specific (sequential) expression of thousands of genes and transposon activation [139]. Indeed, a synchronised model of neonatal spermatogenesis revealed a considerable amount of actively translated transcripts (2345) present in germ cells during spermatogonial differentiation [139], raising the possibility that several different tissue-specific proteins could be expressed by male germline cells. Hence, male germline cells (potentially expressing most of the proteins in an organism) may have the ability to bind to many different extracellular vesicles as well as to different pollutants eventually able to cross the soma-germline Weismann Barrier. Notably, the ability to bind to several antigens/pollutants and/or vesicles/viruses is a peculiar feature of the immune system. In fact, the different immune cell subsets are specialised and cooperating tissues with the ability to recognize different biotic and abiotic (e.g., haptens) molecules. Therefore, male germline cells, sequentially expressing thousands of proteins during their differentiation, may have developed a similar ability. I have already suggested that the immune system elicited by viruses is able to build up new somatic biotechnological tools, which could ultimately be exapted for organism adaptation (for example the mechanism of somatic hypermutation of immunoglobulins was exapted into retrotransposon-guided mutagenic enzymes). It is tempting to speculate that male germline system may possess a similar ability to produce new biotechnological tools. If this is the case, the tools developed in the hypothetical male germline biotechnological laboratory may directly pass on to future generations. Differently, new somatic immune tools have to be transferred to germinal cells before being passed on to future generations. It is known that environmental stress conditions can induced chronic activation of the immune system and inflammation, a condition in which several intercellular messengers such as soluble factors and extracellular vesicles are released by somatic cells. The composition of extracellular vesicles varies depending on (paternal) exposure to specific stress conditions [140], suggesting that they represent a specific response to the new environmental conditions. Since spermatozoa are prone to internalise exogenous material present in extracellular vesicles, it raises the possibility that they could represent a real tool to transfer features acquired by somatic cells to male germline cells for transgenerational propagation. Indeed, it has been shown that extracellular vesicles transmit information regarding paternal environmental stress experience to sperm, altering foetal development [140]. Hence, the new information carried by spermatozoa may represent a paternal “way” to extend “in real-time” to progeny new characteristics acquired during his environmental experience (lifetime). Therefore, somatically acquired genetic novelties may “Lamarkianly” pass to the next generation through male intermediate vectors: a characteristic that resembles Darwinian pangenesis [9,141].

As already mentioned, a direct genome editing mechanism in sperm cells may also be hypothesised. Indeed, the ability to express several different tissue-specific proteins by male germline cells allows them to bind to several different pollutants that eventually cross the soma-germline Weismann Barrier. Like in somatic cells (see Figure 3), the presence of a pollutant interfering with a protein function may lead to a pollutant-mediated upregulation of genome editing mechanisms and its (specific) gene diversification. In line with this hypothesis is the observation that spermatogenesis is particularly sensitive to environmental pollutants [142]. In this case, it can be hypothesised that pollutant-driven gene (allele) diversification in long-lived spermatogonia may induce the generation of a pool of spermatozoa bearing different gene/protein attempts. The spermatozoa bearing proteins that restore the functionality of the protein in the presence of the pollutant (as in Figure 3B) will finally survive and expand (as for B cell clones in SHM), increasing the likelihood to transmit this new characteristic to progeny through a non-Mendelian inheritance. To verify this hypothesis, I have proposed to investigate the occurrence of hypermutation of human killer cell Ig-like receptors (KIRs) in spermatozoa of HIV-1 seropositive patients, as a model of non-Mendelian inheritance. In this regard, it is known that natural killer cells expressing some *KIR3DL1/3DS1* alleles in HLA-Bw4-positive subjects provide protection against AIDS progression [143,144]. KIR3DL1/3DS1 receptors are exclusively expressed by immune cells and in particular by natural killer cells. They are encoded by a rapidly evolving and highly polymorphic locus owning both activating (*KIR3DS1*) and inhibitory (*KIR3DL1*) alleles. The different alleles have different specificity and avidity for HLA-B molecules (the most polymorphic MHC class I), which confer to natural killer cells an inter-individual variability in recognising and killing target cells [115]. In Africa, the continent where HIV continues to pose a major health risk (selective pressure) because pharmacological treatments are still inadequate, it has been shown that KIR3DL1/3DS1 receptors have an unusual selection associated with the expression of peculiar alleles [115]. Therefore, it is conceivable that the *KIR3DL1/3DS1* locus is still evolving in areas in which HIV is still inducing selection. I have already shown how gene “polymorphisms” can be generated by non-random genome editing mechanisms (see for example the somatic Ig hypermutation in B cells); however, in natural killer cells of HIV positive patients there is no evidence of “somatic” *KIR3DL1/3D*S1 “hypermutation”, suggesting that the new *KIR3DL1/3DS1* alleles might be generated in non-somatic organs. Indeed, the HIV tropism for the male reproductive system and the above-hypothesised functional similarity of male reproductive system with the immune system make testis a candidate organ for *KIR3DL1/3DS1* hypermutation.

It is known that under the pressure of the immune system (and of pharmacological treatments), HIV virus rapidly mutates and adapts within the infected individual; nevertheless, up to one-third of HIV-infected infants with vertically acquired HIV infection (from mothers) in Zimbabwe (Africa) has been shown to have a slow disease progression and a small proportion (5–10%) has no symptoms, resembling adult long-term slow-progressors [145]. This observation suggests that, despite increasing viral aggressiveness, some new-borns have developed a substantial resistance to HIV infection perhaps through new protective *KIR3DL1/3DS1* alleles. It is tempting to speculate that the generation of new protective *KIR3DL1/3DS1* alleles could be mediated by a *KIR3DL1/3DS1* hypermutation occurring in the spermatozoa of some HIV-positive patients. Drawing a comparison with Ig-SHM in B cells, it is possible to hypothesise that differentiating male germline cells that express *KIR3DL1/3DS1* alleles with relatively low affinity (and protection) for HIV antigens could undergo *KIR3DL1/3DS1* gene hypermutation with clonal expansion and affinity selection when engaged by HIV viral antigens. In this regard, it has been shown that, like follicular dendritic cells in lymph nodes, Sertoli cells in testis display phenotypical and functional features of antigen-presenting cells [146]. Notably, among the different sperm clones, only those increasing the binding affinity (and protection) for HIV antigens will expand, ultimately enhancing the possibility of transmitting protection from HIV infection to progeny.

Finally, new paternal characteristics acquired directly by germline or through extracellular vesicles from somatic cells may be transmitted to embryos, predisposing offspring to face the environmental stress conditions experienced by the father. However, although this mechanism may account for novelties in alleles of paternal genes, it does not explain the transmission to progeny of novel complex genome rearrangements which require similar changes in both paternal and maternal chromosomes, as in the case of somatic chromosomal translocation. Notably, extracellular vesicles have been shown to contain and deliver not only coding RNAs (or DNAs), but also mutagenic enzymes and regulatory non-coding RNAs, such as retrotransposons [37,114,137]. I have already shown that somatic cells can activate both non-random genome editing mechanisms that target hyper-transcribed genes and their regulatory sequences with specific retrotransposon-guided mutagenic enzymes and non-random retrotransposon transposition among hyper-transcribed genes. It is therefore reasonable to hypothesise that stable introns originated from hyper-transcribed retrotransposons and mutagenic enzymes generated by specific chronic environmental stressors (pollutants/viruses) could be carried by extracellular vesicles from somatic cells to sperm cells and through them to embryos. If this is the case, it is possible that during embryo development the paternal sperm cargo of retrotransposons and mutagenic enzymes could act on both parental chromosomes. In this regard, following fertilization, the DNA methylation marks of both parental gametes are erased [147], rendering the chromatin structure of both parental chromosomes accessible to the genome editing effectors (retrotransposon and mutagenic enzymes) carried by spermatozoa. The sequence-specific binding mediated by the unique sequence of intronic retrotransposons could assure the target specificity of genome editing effectors that ultimately might produce not only gene editing but also changes in regulatory sequences (through retro-transposition and/or editing of regulatory sequences) and rearrangement on both paternal and maternal chromosomes. Hence, somatic extracellular vesicles released into the bloodstream and transferred to sperm cells could bridge the gap between the somatic and male germline cells, ultimately mediating spermatozoa-driven non-Mendelian transgenerational inheritance of novel coding genetic material, non-coding regulatory sequences (involved in epigenetic networks and inheritance) and also chromosomal rearrangements (involved in speciation) stemmed from somatic cells. In this regard, evidence of non-Mendelian inheritance and transgenerational inheritance of acquired characteristics are well documented in several reviews (see [9,11,22,137,138,141]). In these reports, it is suggested that newly acquired traits carried by sperm cargo predispose embryos to cope with new persistent environmental conditions. These insights, if confirmed, could pave the way for the development of new strategies to help future generations to cope with new environmental conditions, for example, by means of new genetically engineered “transgenerational vaccines” that aim to protect progeny by treating young males.

### 7.2. The Fascinating Role of “Female”in the Embryo-Foetal Development and the Revisiting of Haeckel’s Evolutionary Theory: The Involvement of Epigenetic Memories

Several lines of evidence suggest that male germ cells, like viruses for cells (see Figure 1), are crucial for the transmission of novelties into oocytes [9,137,138]. However, every novelty has to be validated for its compatibility with pre-existing and constitutive structures. It is tempting to speculate that the “validation” of novelties is performed during “female” embryo-foetal development. “Female” embryo-foetal development is a fascinating and complex process involving the sequential and finely organised steps of gene expression that is unique for each species. To enable such a complex and finely organised process to occur, the sequence of steps during embryo-foetal development must be somewhere stored. Notably, the DNA is a mere database made of nucleotide sequences forming genes and non-coding regulatory regions, which are totally useless in the absence of a memorised program of coordinated gene expression. Therefore, it raises the question of where the coordinated sequence of gene expression originates. There must be a mysterious “epigenetic memory” that determines a precise sequence of events from zygote to new-born. In this regard, it comes to mind the Haeckel’s evolutionary theory that suggested that the sequence of steps occurred during the evolutionary history of a species (phylogeny) is stored and retraced during the embryo development (ontogeny), i.e., “ontogeny recapitulates phylogeny”.

Is it possible that this “epigenetic memory” originates from past experiences during phylogeny? My answer to this question is yes, I believe that we have to revisit and improve upon Haeckel’s theory. In this regard, it has been observed that organisms of different phyla appear to resemble each other at the phylotypic stage [148], pointing to a common ontogenetic frame that may come from a shared phylogenetic route for a certain period of time during eukaryotic life. For example, the extremely rapid proliferation rate and the relative resistance to ionising radiation (“prokaryotic-like behaviours”) during the early phases of embryonic development of all major animal phyla are suggestive of a common “prokaryotic” origin [132,148,149]. However, it is unclear how so much information could be stored in the “epigenetic memory”. In this regard, it is possible to hypothesise that the starting (environmental/epigenetic) event, i.e., the ovum fecundation by a spermatozoon, could trigger a chain reaction of sequential events mediated by physical interactions between epigenetic (e.g., proteins and RNAs) and genomic (e.g., promoters, enhancers and genes) factors that, according to a program “stored” during phylogeny in an “epigenetic memory”, may lead to the birth of a new individual of the species. It is conceivable that, during phylogeny, both epigenetic and genomic interplays have been continuously modified according to the sequence of environmental changes through the already described mechanisms of transposon mediated non-random genome editing of both genes and regulatory sequences, and retrotransposon transposition. Intriguingly, transcriptional activation of transposable elements and targeted retro-transposition events that reshape the embryo regulatory pathways are common and unclear phenomena that occur during embryogenesis [137,138], further suggesting a similitude between the processes. But what could be the mechanism of phylogeny memorisation?

We now know that chronic environmental stressors can induce biological changes (adaptation) and environmental/external structures in persistent/chronic interaction (chronic stressors) can ultimately be internalised by organisms. Indeed, environmental components, such as pollutants, viruses and microorganisms, can become integral components of more and more complex organisms through a mechanism of “environment internalisation” (see Section 5.2). On the basis of this phenomenon, it is not so unreasonable to suppose that the sequential internalisation of environmental factors into biological structures (during phylogeny) could be “exploited”, during embryogenesis, to reproduce “environmental” conditions able to foster a sequence of biological adaptations similar to those occurred during phylogeny (phylogeny memorisation). Hence, it is possible to propose the incredible hypothesis that, to retrace the sequence of steps that occurred during phylogeny, the embryo-foetal development also has to reproduce the sequence of “environmental” conditions which have led to the species. As already suggested (see Section 4.3.), the epigenetic factors interacting with the genome constitute its “environment” and an “intermediary” in between the external environmental conditions and the genome. Therefore, new environmental stressors experienced during phylogeny would be transformed/transduced into new epigenetic pathways which, constituting the new environment for the genome, would be able to induce new genomic responses. The ability to memorise the sequence of new and successful epigenetic pathways developed during the phylogenetic experience in an epigenetic memory would be able to lead to the memorisation of the phylogenetic process. Notably, the epigenetic-driven genome adaptations and selections that slowly occurred during phylogeny will be quickly retraced during ontogenesis through a domino effect (a chain reaction) that involves an interplay between epigenetic and genomic factors and leads to an elevated time compression (when compared to phylogeny). On the other hand, all organisms would keep “memory” of their past environments and, in this view, they would be like living fossils!

Taken together, these hypotheses suggest that embryo-foetal development would represent a sequential process of biological adaptations, niche constructions and niche internalisation that follows the sequence of ancestral past experiences of the species. The process would constitute the frame of the ontogenetic development and would tend to be independent on the environment in which the mother is located. However, it is also recognised that the ontogenetic processes among different species diverge not only at later stages of their development (as expected from the mechanism of terminal addition claimed by Haeckel’s biogenetic law) but also at earlier ones. In this regard, the trophoblast that was newly added during the evolutionary history of mammals is functionally analogous to maternally derived granulosa cells during oogenesis in birds and possibly reptiles [148], pointing to an environment (maternal tissues) “internalisation” by mammalian embryos. In general, it is expected that the frame of the ontogenetic process could be altered when the mother is subjected to chronic environmental stressors able to reach the “protected” embryo. Indeed, new pollutants/viruses (e.g., Zika virus) or deficiency of essential components that are able to interfere with biological structures involved in specific stages of embryo development can potentially induce a stage-specific remodelling (in search of a new adaptation) of the phylogenetic frame. In this regard, there is evidence of maternally mediated transgenerational effects, for detailed information see [9].

Notably, the possibility that the phylogenetic route might constitute the frame of the ontogenetic process can provide a rationale for some weird aspects of embryo-foetal development. For example, during animal development, several apoptotic events, occurring after cell proliferation stages [150], are active processes that require an unexplainable waste of biological energy. Indeed, during embryogenesis, various structures that are needed at one stage of development are later removed by apoptosis and many organs overproduce cells that are then eliminated by programmed cell death. Programmed cell death is an important process because it sculpts the organs by eliminating cells that over time of animal development, at certain point, become non-functional [150]. Indeed, apoptosis is a sort of a quality-control process that adjusts the correct number of cells and selects the most adequate cells for the organism development. Notably, this phenomenon is a sort of mass contraction that resembles the competition and contraction phases occurring after immune cell expansion during either the final steps of immune response or the selection steps of adaptive immune cells. Indeed, developing B and T lymphocytes that either express potentially dangerous self-reactive receptors or fail to express potentially useful antigen-specific receptors are eliminated by apoptosis. This selection is thought to depend on a competition process for a limited amount of survival signals coming from neighbouring cells (environment) that finally ensures the survival of the most adequate cells [150]. Notably, a similar phase sequence (expansion, competition and contraction) driven by the amount of nutrients was previously described for the generation of new (cell) species (see Section 5.4), suggesting that the proliferation and apoptosis phenomena during embryogenesis might reproduce a sequence of cell speciation, expansion and selection occurred during the evolutionary history of the species (phylogeny). Therefore, in the absence of perturbing factors, the initial trigger of sperm entry into the ovum would switch the developmental program on, starting a cascade of events (a domino effect) that retrace the phylogenetic steps. Similarly, the cell-mediated immune response after an infection (initial trigger) sequentially activates different immune cell subsets in an order that follows the ontogeny of the immune system itself: first innate myeloid cells, then innate lymphoid cells and only at the final step the adaptive immune cells responsible for pathogen eradication.

Finally, sexual differentiation represents an altruistic symbiotic solution in which the males (like viruses: “virile as viral”) are mainly involved in the transmission of novelty, fragmentation/harmfulness and variability while the females (like cells) in transmission of history, integration/complexity and memory (in this regard, in females there is also evidence of telegony and foetal micro-chimerism, see [9,151]). The power of sexuality likely originates from the complementarity of biological abilities; however, the increase of biological complexity and “variance” is now blurring the distinction between genders (e.g., in human beings), further increasing heterogeneity and adaptability.

## 8. Conclusions and Future Perspectives

### 8.1. Non-Random Genome Editing Mechanisms: A Link between Environmental Changes and Eukaryotic Biological Novelties

In the present work, I have explored several lines of evidence that support the existence of molecular mechanisms of non-random genome editing developed and exploited by human cells and, more generally, eukaryotic cells to respond to new and chronic/stable environmental conditions.

When the cell environment is sufficiently stable and cell reversible/epigenetic/homeostatic mechanisms are sufficient to respond to normal molecular fluctuations the cell eukaryotic genome is adequate. However, in the face of strong and chronic environmental changes (new pollutants/microorganisms and/or new molecule concentrations), the mechanisms of non-random genome editing would be induced to “surgically” produce several eukaryotic gene attempts (gene plasticity) and new regulatory sequences (“epigenetic” plasticity).

Finally, as the environmental changes become relatively stable, the best biological solutions would be selected and stably memorised in the DNA, accounting for both the genetic and the so called “epigenetic” inheritance. Non-random genome editing mechanisms may represent a link between environmental changes and eukaryotic biological novelties, creating a dynamic world where genomic plasticity, together with symbiosis and horizontal gene transfer (among species in persistent interaction) are common consequences.

The existence of these genome editing mechanisms would establish the centrality of the environment, of which life is now a fundamental part, constructing specific niches/habitats (environmental plasticity). Indeed, the environment can influence biological structures at different levels, inducing both their variation/transformation and selection. However, only molecules (e.g., pollutants) that penetrate and reach cell compartments may generate cell environmental changes able to induce “molecular” DNA mutagenesis. Notably, tissue phenotypic variations induced by tissue-specific gene mutations create a cascade of events involving neighbouring cells that sequentially affects organs and the fitness of the whole organism. Therefore, the outcome of the mutagenic responses in an organism does not depend only on the specific mutation but also on the response of the neighbouring tissues (the environment around the mutated cells), which can be different in different organisms (e.g., some chromosome translocations can be either pathological or harmless). Similarly to an external cell stimulus that induces an “outside-in” signal, an environmental perturbation produced by exogenous “penetrating” molecules (pollutants) can induce an “inside-out” organismal response that yields a new environmental condition at outer levels (niche construction/environment plasticity), which will depend not only on the type of pollutant (stimulus) and of organism (cell) but also on the starting environmental condition (the environment from which the cell stimulus starts).

### 8.2. A Symbiotic Reconciliation of the Darwinian and Lamarckian Perspectives

The described mechanisms of genome editing perfectly match those whose existence I have “madly” hypothesised in the first part of this paper to justify a body of unexplainable “weird” evidence regarding eukaryotic species. Such mechanisms are activated “on demand” by novel environmental pressures, and they non-randomly/“Lamarckianly” “target” a small number of hyper-functional/hyper-transcribed and environmentally inadequate genes (codifying exons) or regulatory sequences (introns, promoters, enhancers, miRNA) in which they create random mutations/novelties. Random mutations (novelties) in a selected gene are consequence of the functional (ab)use of a specific gene (hyper)transcription (as hypothesised to occur in hyper-activated, “hyper-functional” organs by Lamarck) and lead to phenotypic plasticity. The acquired phenotypic variants/novelties provide raw material for Darwinian natural selection, which will then determine the survival of the most effective version of genes and regulatory sequences (genomic features) in the cell environment (first level of selection). Then, the most effective version of cell/tissue bearing the new genomic characteristics will be selected by the organism environment in the organ compartment (second level of selection). Hence, it appears there are multilevel environments and multilevel selections (see later Section 8.5 for further details). In this regard, it is likely that in new stable environmental conditions, eukaryotic organisms have developed mechanisms through which some of the new and successful genomic and phenotypic features produced by non-random genome editing mechanisms in somatic cells could be passed on and inherited by the progeny of a species (third level of selection). In this regard, spermatozoa from virtually all animal species have been shown to have a particular feature, i.e., the ability to spontaneously take up somatic extrachromosomal nucleic acid information present in extracellular vesicles (the gemmules of Darwinian pangenesis, eukaryotic “plasmid-like” forms of horizontal and vertical genetic transmission), which may be delivered to fertilised oocytes and embryos [9,137,141] (see Section 7.1). On the other hand, the male novelties have to be validated and selected for its compatibility with pre-existing conditions and this is likely performed during “female” embryo-foetal development, which determines the “integration”/acceptance (environment internalisation) or exclusion/refusal of male “advances” (see Section 7.2).

Genome evolution appears to be driven by environmental pressure and mediated by different evolvability mechanisms, from Darwinianly random in first and simple biological structures (through for example error-prone nucleic acid replication in viroids) to those that are Lamarckianly orchestrated (through environmentally induced non-random genome editing mechanisms) in (higher) organisms owning high-fidelity replication mechanisms. Altogether, these considerations “neotenically” renew/rejuvenate the old Lamarckian and Darwin’s Pangenetic theories and ultimately eliminate the conflict (if any) between the Lamarckian and Darwinian perspectives.

### 8.3. Non-Random Genome Editing Mechanisms Reconcile Gene-Centred and “Ecological” Theories of Evolution: Thinking of a New “Hyper-Modern” Synthesis of Evolutionary Theory, Eco-Evo-Memo-Poly

The appearance of life on Earth has accelerated the environmental changes (environmental plasticity), leading to environmental evolution and production of novel environmental heterogeneities through a life-mediated niche construction. This in turn drove biological structures to evolve and create novelties and higher levels of aggregation and complexity, which in turn, in an ongoing cycle, produced new environmental products. In general, chronic environmental stress conditions in defined geographical areas (ecosystems) produce struggles for survival that rapidly induce the development of new biological tools and species (developmental plasticity or clades at different levels of complexity), which locally determine temporary periods of stability (“stasis”). Notably, non-random genome editing mechanisms have allowed the storage of more information in the DNA database (large eukaryotic genomes) while still maintaining its flexibility and adaptability to environmental changes. Therefore, these mechanisms have likely allowed the development of higher organisms in the presence of rapidly adaptable microorganisms and, more generally, in Earth’s changeable environment. Since evolutionary theories cannot disregard both the environmental context and the genetic mechanisms, the non-random genome editing mechanisms that I have described may represent the missing link between ecological and gene-centred views of evolution. Indeed, these non-random genome editing mechanisms may provide the molecular genomic basis for the rapid biological plasticity described both in punctuated equilibrium by Gould and Eldredge [19] and in “ecological” views of evolution [11,16,17,18] and finally, effectively reconciling gene-centred and “ecological” theories of evolution. It is for all of these reasons that I see the need to develop a new “hyper-modern” synthesis of evolutionary theory, in which Earth’s changeable environment (Eco) (especially after the appearance of life) is the starting point that, cyclically driving the biological evolution (Evo) and memorisation (Memo) of the most adequate living forms, ultimately aggregate them (Poly) into increasingly complex organisms, holobionts.

An example of the central role of environment and the strict relationship among environmental changes (Eco), biological acquired mutation (Evo and Memo) and symbiosis (Poly) is well represented by the interplay between malaria parasites and haemoglobin (Hb) variants in human red blood cells [152]. In this regard, geographical distributions of Hb variants known to confer protection from malaria overlap with those of malaria parasites [153], indicating their strict cause-effect relationship. It is indeed conceivable that the appearance of malarial parasites in specific geographical regions started to threaten the survival of human beings by destroying their erythrocytes and that this environmental pressure had ultimately triggered human adaptation responses (e.g., Hb mutants), thus justifying co-evolution of host bearing Hb variants and life-threatening parasites. However, can we hypothesise the involvement of non-random genome editing as mechanisms underlying this interplay? Notably, gene mutation cannot be induced in mature enucleated erythrocytes, therefore how can parasites trigger host Hb mutations?

Non-random Hb gene mutations should occur in the sites of erythropoiesis during erythroid Hb gene hyper-transcription, i.e., yolk sac/fetal liver in human fetus or postnatal bone marrow [154] and they could be indirectly induced by the increase rate of erythropoietic proliferation due to malaria-induced erythrocyte destruction. Alternatively, a direct induction of Hb hyper-transcription is difficult to hypothesise. Indeed, when sporozoites are injected by mosquitos into the human bloodstream, they travel to the liver where they mature into hepatic schizonts. Eventually, the rupture of mature schizonts releases thousands of merozoites into the bloodstream and each merozoite is capable of invading red blood cells causing the symptoms of malaria. During this process, a fraction of merozoites differentiate into the sexual stage, called gametocytes. However, only mature forms of gametocytes (and not immature ones) are present in the blood circulation, suggesting an extravascular site of gametocyte development. In this regard, an interesting study has recently revealed that gametocyte development mainly occurs in the human bone marrow where merozoites, infecting erythroid precursors, undergo development into mature gametocytes before re-entering the bloodstream [155]. It is therefore possible that gametocytes formed in erythroid precursor cells and accumulated in the human bone marrow could induce not only erythroid proliferation but also hyper-transcription of erythroid proteins that may lead to erythroid gene mutations. In this regard, in malaria infections, different stimuli (in particular, low oxygen tension consequent to red cell killing) can induce erythroid cell proliferation and different erythroid gene hyper-transcriptions. Therefore, non-random genome editing mechanisms may induce gene mutations involving not only haemoglobins but also other “bottleneck” genes, finally justifying different malaria-protective red cell variants (haemoglobin mutants, enzymopathies, O blood group protection and acquired iron deficiency [152]).

Regardless the mechanisms of induction of different malaria-protective red cell variants, epidemiological evidence indicates that heterozygosis for both HbC and HbS variants influences pathogenic processes, leading to non-severe clinical malaria outcomes and patient survival. However, characterisation of asymptomatic carriers of Hb mutants indicates that the two Hb variants differently influence the reservoir of infection. HbS heterozygous is associated with reduced frequency of malaria parasites, whereas these parasites were frequently detected in HbC heterozygotes (pathogen/environment internalisation) [153], indicating that malaria parasites are less pathogenic and can cohabit with hosts carrying HbC. This last phenomenon appears to be an initial (pre)symbiotic relationship (Poly) between malaria parasites and heterozygotes carrying HbC mutant allele, which, however, promote malaria transmission. Indeed, these subjects are healthy carriers that can transmit malaria disease and kill subjects carrying normal Hb alleles, i.e., subjects carrying genomes inadequate to counter the environmental challenges (malarial infection). Differently, host microRNA variants present in HbS-containing erythrocytes have been shown to contribute to resistance against *Plasmodium falciparum* by forming chimeric host-parasite mRNA transcripts which result in translational inhibition of parasite protein synthesis [156]. The presence of host microRNAs able to bind to parasite transcripts strongly suggests integration/internalization and domestication processes of eukaryotic protist DNA sequences into host genome that resemble the previously described viral and bacterial domestications (see paragraphs 2.1 and 3.5, and [11,16,26]). On the other hand, this evidence suggests the origin of microRNAs as tools of host defence against complex eukaryotic pathogens (e.g., Plasmodium protists) that were developed by “domestication” of pathogen DNA sequences. Interestingly, phenomena similar to those occurring in HbC and HbS subjects can be observed in long-term non-progressors (subjects with high viral load) and spontaneous controllers (subjects with undetectable or low viral load) of HIV infection [65] and possibly in SARS-CoV-2 infected subjects [157], suggesting that both “pre-symbiotic” intermediates (e.g., in HbC subjects) and forms of domestication and exaptation of pathogen sequences into host defence functions (e.g., in HbS subjects) may be common stages of pathogen-host interplay. Regarding the global pandemic threat of SARS-CoV-2, the chronic engagement of angiotensin-converting enzyme 2 (ACE2) cell surface protein for viral entry is expected to induce ACE2 gene “hyper-transcription” [158] and ACE2 hyper-production was recently documented [159]; consequently, its hypermutation by non-random genome editing mechanisms is also expected, a prediction that should be verified in patients with chronic SARS-CoV-2 infection.

Now, the sequential evolutionary steps of malaria-host interplay can be briefly summarised as follow. First, the endemic malarial environments (Eco) localised in specific geographical regions and in precise lag of time (ecosystems) determine non-random biological responses in infected subjects, leading to rapid evolution (Evo) of specific Hb gene variants (clades) and/or horizontal genomic transfer between the two struggling species (usually, but not exclusively, from the simpler to the more complex species). Then, the survival of red blood cells bearing the fittest variants (natural selection) determines the genomic mutants that will be inherited and memorised into the DNA (Memo) of surviving organisms. Finally, some successful solutions (HbC/HbS) produce an environment internalisation i.e., a symbiotic aggregation or horizontal genomic transfer of pathogen sequences into host genome (Poly). Notably, the integration and domestication of malarial plasmodium DNA sequences in HbS-bearing cells (as well as of viral sequences into infected cells) provide new biological tools that in the future could be exploited to face the perpetual environmental cha(lle)nges present on Earth in a fashion not related to the original host defence function (exaptation). Finally, the evolutionary process, passing through sequential steps (Eco-Evo-Memo-Poly) that recur cyclically, is able to progressively build up higher levels of biological aggregation and complexity, which are exemplified by the eukaryotic (higher) organisms (both animals and plants). On the other hand, during this process of biological aggregation, living organisms progressively “eat” part of abiotic/inorganic environmental components through a mechanism of environment internalisation.

I believe that this new theory of evolution based on molecular mechanisms represents a serious and organic effort to unify the evolutionary theories; it sheds light not only to several unclear aspects of microevolution but it might also provide a foundation for the understanding of multimutation leaps typical of macroevolution.

### 8.4. Multilevel Biological Organisations and Multilevel Selections: A “Philosophical” Structuring of Life

The biological system as whole appears to be formed by multilevel communicating nested subsystems in which the higher level of biological complexity/organisation constitute environments for the neighbouring lower levels. The levels of biological complexity/organisation go from molecule to ecosystem levels passing through intermediate levels (such as virus, cell and organism levels) and undergo a multistep adaptation and selection process. Between neighbouring levels of biological complexity there are inter-level borders/surfaces (e.g., cell membranes and organism surfaces) that through specific bidirectional “transmitters-receivers” interactions mediate inter-level communications and cascades of biological adaptations and selections either bottom-up or top-down. In this regard, the multilevel selection theory [160] has already proposed the biological system as a matryoshka-doll, in which natural selection operates at multiple levels of the biological hierarchy and also that adaptations at lower levels influence higher levels. Indeed, the degrees of freedom in adaptive solutions/selections to environmental challenges exponentially grow with the level of biological complexity; however, the adaptive solutions/selections at lower levels restricts the adaptive possibilities and the real degrees of freedom at higher levels. Since lower (e.g., molecular) levels of biological complexity appeared earlier than higher ones, the latter and younger levels are built from the earlier and “elder” ones, which, on the other hand, have been selected in past and different Earth environments (selective conditions). Among the older adaptive solutions/selections, there are some that will never change (e.g., the genetic code, the number of cervical vertebrae in a giraffe etc), while other “epigenetic” adaptations may reverse. However, the biological adaptations that are “fixed” in the past will bias the subsequent biological adaptations restricting the possible adaptive solutions to new emerging environments. For example, the RNA was likely the most stable molecule produced in the environmental conditions of the primordial soup (but not in the current environment) and this aspect was likely determinant for the subsequent biological evolution (see later, on the origin of biomolecules, Section 8.6), by limiting the subsequent adaptive possibilities (degrees of freedom). That is to say that the initial “imprinting” (determined by the initial environment) can be crucial in determining some of the future developments. Indeed, the new biological “buildings” produced under the “stimulus” of new emerging environments (either in the past or in the future) appear to come from the reuse and the redistribution (exaptation) of some old and stable “bricks”, possibly in a more and more stable manner (for further argumentation, see Section 8.8).

As already mentioned, the environmental changes produce adaptation and selection at each level of the biological hierarchy. In the case of appearance of new environmental molecules/pollutants (newly generated or migrated) able to interact with molecular biological structures present in a cell/tissue, it will be induced an initial cell/tissue response characterised by cell molecular modifications (molecular level interplay). New genes/proteins and regulatory sequences (molecular levels of biological complexity) will be selected to meet the needs of the cell/tissue in the presence of the pollutants and the new molecular solutions will determine the survival or not of the cell/tissue. However, the surviving cell/tissue will be different in the different organisms of a species depending on both their genome and their phenotype (e.g., phenotypes determine the type of interactions with pollutants). Subsequently (bottom-up), among the different surviving cell/tissue solutions in the organisms, only those that meet the needs of the organism will survive and be selected. On the contrary, organisms bearing environmentally inadequate genomes will succumb because they will develop an organism mediated tissue cell death (e.g., autoimmunity/tissue rejection) or a cell mediated diseases (e.g., cancer/graft versus host disease). Among the adequate genetic solutions present in the different organisms, only those that will be transferable (if any) to progeny will be inherited (i.e., selected and “memorised” by the species). However, in the case of appearance of new biological entities belonging to intermediate levels (newly generation or migration of viruses/cells/organisms) and able to produce an imbalance at that specific intermediate level of complexity (because of new intra-level biological interactions and/or new intra-level competitions for nutrients and struggles for survival), it will be induced an initial adaptation and selection at that precise intermediate level of biological complexity. Subsequently, it will be generated a cascade of adaptations not only bottom-up to species level (e.g., new symbiosis developing new organism functions/skills) but also top-down to molecular level (e.g., new molecular domesticated structures that allow to transform pathogenic into symbiogenetic interplays). Finally, selection can occur at even higher levels of complexity and not only at inter-species levels (e.g., during prey-predator dynamics) but also at intra-species levels (e.g., during human wars) between different cultural ecosystems.

Ultimately, multilevel biological selections of the most “environmentally” adequate “solutions” are produced by the exclusion of conflicting and death-producing biological entities and the promotion of the more stable “symbiogenetic” structures. Interestingly, in some cases (e.g., eusocial insect colonies [160], the biological traits “for the good of the group” (upper biological level) may prevail against those for the good of the individual (lower biological level). The altruistic behaviour of a level of biological organisation in favour of the upper level is indeed a common aspect. For example, cell apoptosis is an active altruistic process of an individual cell that is activated in order to preserve an organism, a cell community, in which the cell resides. Similarly, an individual of an animal species can perform “self-sacrificing” behaviours in favour of the survival of the rest of the species. For example, alarm signals emitted by a prey usually function to both attract predators and warn nearby preys, giving them a better chance of escape. The accidental condition of being the first to see a predator determines which prey will be killed by the predator. During biological evolution, the “altruistic” mechanisms that preserve the upper levels of biological organisation are likely necessary for the survival/existence of the higher levels and are therefore selected top-down at each level (possibly with the exception of the highest). Indeed, the selfish trait of a cancer cell (as well as that of a virus or of a predator) creates conflicts that threatens the survival of the community of cells (organism/“holobiont”) and is consequently lost with the death of the organism (as well as with death of the virus infected cells or with the destruction of the ecosystem by an aggressive predator). In this regard, the selfish traits of human beings are creating conflicts (as a harmful virus/cancer/predator) that are threatening the survival of the community of ecosystems (“megabionts”) on Earth. This situation will inevitably lead to either a progressive and fast exclusion of selfish eukaryotic organisms and selection of altruistic traits or a massive extinction, as occurred with dinosaurs.

Nevertheless, new conflicts (e.g., between new viruses and immune systems) that are continuously created by new environmental conditions are important to push the development of new technologies and complexities (see Section 4.6 and later Section 8.7). However, if we are aware of this, now we could decide to develop new technologies trying to avoid conflicts, as we are now countering viral infections/conflicts with targeted vaccinations.

### 8.5. Biological Systems as Fractal Systems with a Nested “Matryoshka Doll” Structure

Self-similar structural and functional patterns across different levels of organisation/complexity are typical of fractal systems, which also exhibit a high level of (self-)organisation and iterative pathways [161]; all features that give living forms the ability to adapt to a changing environment, maximising fitness. My general impression is that biological systems are fractal systems with a nested “matryoshka doll” structure. At different levels of organisation/complexity, biological entities are formed from a sequential aggregation of slightly different basic modules (“monomers”) that together form aggregates (“heteropolymers”), which in turn become basic modules for superior aggregations that may have patterns resembling those of lower levels although with higher levels of complexity (see Figure 1). In this regard, we can imagine a scenario in which different viruses become basic modules (“monomers”) whose sequential aggregation leads to the formation of different cell types (“heteropolymers”). In turn, replication of different cells (“monomers”) forms tissues (“homopolymers”) that become basic modules for organ (“heteropolymers”) formation and different organs (“monomers”) become basic modules for organism (“heteropolymers”) formation. Finally, different “homopolymers” of replicating species become basic modules for ecosystem (“heteropolymers”) formation. However, some of these biological “aggregates” are incapable of autonomous life (e.g., a virus, an organ or a species) and represent fractions/fractals/compartments of the autonomous biological entities, which can carry out all the vital functions (e.g., a cell, an organism or an ecosystems).

Notably, a similar scheme/approach can be applied to atomic and subatomic elements and perhaps even to celestial bodies, all distinguishable in species (“heteropolymers”) formed by the aggregation of different “monomers”. For example, basic modules of protons, electrons and neutrons (three different “monomers”) aggregate into atoms (“heteropolymers”), and atoms (as “monomers”) aggregate into molecules (“heteropolymers”). During these processes, the matter progressively increases its aggregation. Can this process of matter aggregation be used to justify the selection of specific biomolecules on Earth?

### 8.6. On the Origin of Biomolecules and Virus-Like Structures

The starting point of the history of life can be traced to the particular environment on Earth, namely the presence of huge amounts of water in which myriads of molecules could be synthesised (a primordial soup). In this regard, it has recently been hypothesised that nucleic acids, amino acids, and lipids were produced from a pair of simple compounds abundant on early Earth [162]. These three classes of biomolecules may have given rise to aggregates able to produce a primordial form of life. Importantly, aggregation mediated by weak inter- and intra-molecular bonds stabilises molecules and excludes them from further chemical reactions, creating a progressive selection of the most stable molecular aggregates. In this regard, during the hypothetical stage of the “RNA world”, the formation of stem-loop structures of RNA through intra-molecular hydrogen-bonding interactions (A-U and C-G) would have generated the more stable double helix RNA and the non-enzymatic template-directed RNA synthesis [163]. Indeed, the binding of a complementary oligonucleotide under the direction of a pre-existing RNA sequence can foster both phosphodiester bonds between nucleotides and the preferential recruitment of cytosines and guanosines because of their three stabilising hydrogen bonds. A possibility that might justify the generation of both CG-rich viroid genomes (the first virus-like structures of the “RNA world” [2,3,164]) and CG-rich promoter/enhancer regions, (fossil forms of anciently inserted viral sequences). Moreover, it can be hypothesised that during the “RNA world” stage, some amino acids were able to bind to repetitive RNA sequences through weak molecular bonds (such as the amino acids in conserved regions of APOBEC or Cas proteins able to bind to viral repetitive RNA transcripts). This may have led to “selection” of amino acids with a specific three-dimensional structure (e.g., L-enantiomers) and also fostered peptide bond formation (peptide synthesis) between amino acids coming in close proximity by binding to repetitive RNA sequences. Primordial peptides able to form RNA-peptide complexes gave a higher chemical and structural stability to RNA and stable RNA–peptide aggregates (“heteropolymers”) may have generated the primordial “naked” viruses (without envelope). The “biological bricks” to build the primordial viruses were supplied by their passive diffusion in the primordial soup (primordial soup as a host) until competition for biological bricks occurred. In this second phase, molecules capable of ”stealing” amino acid and accelerating amino acid recruitment and proximity such as tRNA-like (parasite-like mobile elements), may have been selected. In this regard, tRNA-like structures capable of specific aminoacylation have been found in a number of plant RNA viruses, and some large DNA viruses have been shown to produce their own tRNAs [165,166]. It is therefore possible to imagine that the back and forth horizontal transfers of some of these viruses might have given rise to “domesticated” eukaryotic tRNA molecules. Finally, aggregation of RNA elements coated with amino acids/peptides and able to recruit/steal amino acids from the primordial soup may have generated error-prone replication and “translation” systems (random formation of first ribosome-like structures). Error-prone primordial forms of ribosome-like structures were able to generate myriads of different molecular attempts (both nucleic acid sequences and peptides) that were continuously tested and selected. The production of a huge number of different molecules was highly efficient to explore a new fitness scenario that finally developed structures/aggregates with higher both stability and capability of reproduction. Of note, viral genes mainly code for proteins able to bind to its own nucleic acid code, this feature favours a “viral” structural stabilization because the coating of proteins stabilize and protect the nucleic acid code, finally generating a self-reinforcing feed-back loop. Therefore, during the RNA world, among myriads of ribosome-like attempts, those able to produce peptides with affinity for their own nucleic acids and leading to stable nucleic acid-peptide copies (i.e., naked virus-like structures) have been positively selected. Indeed, this is not surprising if we think that spontaneous chemical reactions lead to chemical products that are more stable than their reactants, and biomolecules and viruses are chemical products originated from the primordial soup reactants.

Finally, RNA–peptide aggregates with affinity for lipids present in the primordial soup may have generated different RNA–protein–lipid “viral” aggregates (“heteropolymers”), giving rise to enveloped viruses. With time, the RNA catalytic activities and coding functions were replaced and improved by enzymatic proteins and the more stable DNA molecule, respectively. DNA is a negatively charged nucleic acid with high affinity for the positively charged basic amino acids lysine, arginine, and histidine, which are the main components of histone proteins. In the eukaryotic nucleus, DNA is wrapped around histones and condensed into chromatin, in this way the genetic material is better stored and conserved, thus ensuring high-fidelity DNA replication typical of eukaryotic cells [1,2].

After the generation of a myriad of viruses, they started to compete and aggregation into primordial cells was likely a successful solution. As already hypothesised (see Section 3.5), cells may represent communities (heteropolymers) generated by sequential integrations of viral monomers composed of operon modules and viral repetitive sequences with protein-binding functions. Similarly, organisms (heteropolymers) can be thought of as sequential aggregations of organ modules (monomers). Notably, all these modules (except when they become selfish and predatory) can be seen as “biological bricks” that have been domesticated/educated to fulfil the functions of the higher (host) level.

The primordial soup that initially supplied viruses with “biological bricks” for their replication (primordial soup as a host) was subsequently substituted by the advent of biological entities able to plan the “robbery” of the biological bricks from the environment, i.e., tRNAs, cells and organisms, finally moving from primordial soup to cells and organisms as viral hosts.

Although the primordial soup is the plausible place for the origin of life on Earth, there is evidence that some forms of life could have been generated in several different environments of the Universe, giving rise to an alternative panspermic viewpoint [9]. In this regard, the generation of life may not be necessary geocentric (an exclusive Earth matter); however, the multitude and, in particular, the complexity of living organisms developed on Earth through a Lamarckian mechanism is likely unique or extremely rare. At the moment, a single species has been able to adapt the environment to its needs and thus to colonize the whole planet starting from a single (African) birthplace. In the future, if humans will be enough intelligent to survive to their own wastes (e.g., CO_2_), I think that they may be able to colonize the extra-terrestrial space and to export Earth biological complexity outside the solar system. Indeed, humans have already been able to create living tunnels until to the Moon crossing life-incompatible spaces, thus starting a “geospermic” process.

### 8.7. The Evolution of Viruses as Migrating “Organelles” of Cells: Implications for Human Brain Evolution

The existence of retrotransposon-guided proteins (mutagenic enzymes, multifunctional proteins, transcription factors etc) suggests that viral repetitive non-coding and apparently “junk” DNA elements, accounting for almost half of the human eukaryotic genome, may not be useless “selfish” or even parasitic elements, but rather crucial elements of the sophisticated eukaryotic cell still involved in mediating response/adaptation to environmental challenges. Nevertheless, the majority of viral structures maintain their cell-killing and migrating features that exert strong selection pressure on their host organisms. How is it possible to reconcile virus cell-killing ability with virus domestication?

In this regard, the hypothesis that viral structures represent migrating “organelles” of cells may help in elucidating this issue. Indeed, in line with this hypothesis are the observations that genetic content can flow not only from virus to host cell, but also from host to viral genomes, as exemplified by host-derived: (1) stress-induced ERV reactivation [105,106]; (2) viral defence mechanisms; (3) viral capsid proteins and; (4) plasmids coding for viral replication proteins [33,167,168,169]. These observations both justify the viral “escape hypothesis” [167,168] and exemplify the “guns for hire” concept developed by Koonin and collaborators [33]. However, the back and forth horizontal transfers of genomic components between viral elements and cellular systems also reveals that: (1) an initial cause (e.g., viral infection) produces an effect which influences the starting cause through feedback loops (viral genome editing), making it difficult to determine what the original cause and the cause-effect relationship of the phenomenon are; (2) each migration in a new environment does not induce a mere and static genomic shuttling but also a genomic evolution/adaptation mediated by both random viral mutations and non-random cell-mediated genome editing mechanisms. These phenomena are expected to cyclically generate either locally and temporary inactive (non-interacting) viral elements or new parasites or new symbiotic elements. However, since symbiosis among biological elements is a more stable solution, a progressive aggregation and complexity (holobionts) is expected over time. Indeed, symbiosis is a successful biological strategy that is fostered by natural selection at different levels (virus–cell, male–female, familial aggregation, community aggregation and species aggregation in ecosystems).

Some viral structures may therefore be seen as migrating “organelles” of cells that, going abroad to other biological “countries” (tissues of other organisms) undergo new (“stressful”) conditions (e.g., new host immune pressure shaping virus diversity), and learn and acquire new “skills” (e.g., host-induced new viral immune evasion strategies and/or new structural or replication protein induced by host-driven domestication). These new structures can either kill (negative selection) obsolete and no longer adequate tissue cells (carrying genomes unable to manage them) or provide new tools to some tissues with a predisposing genome that will be tested in facing the challenges of a continuously changeable environment. In this regard, I have already described that the different APOBEC mutagenic enzymes might derived from pathogen domestication and that they have been exploited to counter the burst of several types of ancient tissue-specific retroviruses. These retroviruses in turn would be domesticated into different Alu and LINE subfamily retro-elements. Notably, the number of *APOBEC3* paralogs in humans (seven) is particularly high if compared with mice (only one) [36], indicating that extensive retroviral infections might have triggered the duplication and divergence of *APOBEC3* genes in humans. Moreover, APOBEC mutagenic activity (C to T conversion) have been shown to increase retrotransposon diversity and therefore the probability of their exaptation for novel functions, finally boosting genome evolution in vertebrates [170]. However, in humans, there is prevalence of another (domesticated) mutagenic activity, the A to G conversion mediated by ADAR enzymes [171]. When compared to other mutagenic activities, this conversion is significantly higher in humans than in mice, suggesting that elevated ADAR activity might be primate specific [171]. On the other hand, the prevalence of Alu elements in the human genome indicates that most Alu sequences are subject to ADAR editing [171,172]. Since ADAR activity is most prevalent in the brain tissue [171,172], it is likely that ADAR editing of Alu sequences may be involved in brain functions. Indeed, the brain is a genomic mosaic and retrotransposon mobilization is believed to be an important source of neuronal genomic diversity; a genomic plasticity that may underlie neuronal plasticity [173,174]. Finally, the neuronal gene *Arc*, essential component for information storage in the mammalian brain, has been shown to encodes a protein that forms virus-like capsids and exhibits properties similar to retroviral proteins [175]. Indeed, virus-like capsids of Arc protein carrying its mRNA are released from neurons in extracellular vesicles and this phenomenon causes the release of more Arc protein from other neurons through a “viral” domino effect [175], suggesting a link between virus infections and development of neuronal plasticity. For the above-mentioned observations, it is tempting to speculate that human intelligence might come from extensive ancient infections of Primate “Lineage”.

### 8.8. Conclusive Chemical and Physical Considerations on Life: The Big Bang of Life as a Gravitation-Like Force of Aggregation, a Gravitational Singularity with a Black Hole-Like Behaviour

Biology is largely regulated by physical and chemical rules and throughout the paper I have highlighted similarities between the animated and inanimate worlds. In this regard, it is interesting to note that the expansion and contraction phases that characterise the expansion of a new species or the response of the immune cells to viral infections (see Section 5.4) resemble the phases of a chemical reaction in which the biological species (cells or organisms) are both reactants and final products. When there are sufficient amounts of contacts (activation energy) between a biological species (first reactant) and nutrients (second reactant), the process starts, intensifies, reaches a maximum rate of expansion and then decreases (a contraction phase) when the nutrients (the limiting reactant) is insufficient for the needs of the species that starts to compete for it. Finally, similarly to a chemical reaction, a dynamic steady-state equilibrium between products and reactants is achieved. Such a balance depends on both the rate (if any) of new intake of the nutrients/limiting reactant in the system and on the inhibition (if any) of “waste” products coming from the metabolic/reactive process. However, similarities between biological and abiotic processes are not limited to chemical aspects.

I have already shown that living systems spontaneously tend to aggregate in more and more complex systems which progressively internalise part of abiotic environmental components; however, living systems tend to internalise and aggregate matter (and energy) at several different levels. For example, the biological system as a whole absorbs energy from the extra-terrestrial environment (mainly UV photons from solar radiation) for its expansion and aggregation purposes, giving very little (mainly low-energy infrared radiation) back to extra-terrestrial environment (considering that E = mc^2^, the famous Einstein equation). Moreover, all living systems, catalysing spontaneous chemical reactions, accelerate the formation of more stable products from reactants. However, to produce more stable molecules from chemical reactions there has to be a redistribution of atoms in a way that reduce the repulsive (or increase the attractive) forces among them, finally leading to a higher stability of the products. As consequence of the reduced repulsive (or increase attractive) forces among atoms, the volume of the products should substantially decrease when compared to the volume of the reactants. Indeed, the majority of spontaneous reactions produces a volume contraction (although the amount of matter/atoms is always the same!), as for example when two oxygen atoms strongly bind together with a covalent double bond to form O_2_ molecule (this aspect will be discussed in detail in a future paper). That means that life, accelerating spontaneous chemical reactions, not only aggregate but also compact matter, progressively increasing matter density. Indeed, biophysical data surprisingly indicate that atoms inside proteins, at interfaces of protein–protein complexes and at the protein–DNA interfaces are as closely packed as in crystalline solids [176,177], suggesting that matter inside living organisms as well as that inside celestial bodies in the Universe may undergo a local compaction process and increase of density. Indeed, similar to gravitation, life works like a force of matter aggregation and compaction that progressively integrates the elements that start to interact with it through the weak intermolecular bonds of complementary stereostructures. Interestingly, the progressive increase of molecule (e.g., protein polymorphisms) and organism (e.g., number of species) heterogeneity/variability produced during life processes, also increases the probability of interaction and binding to new molecular stereostructures (e.g., pollutants) and therefore exponentially accelerates the aggregation processes. Therefore, life, capturing photons, accelerating spontaneous reactions, transforming abiotic into biotic elements and increasing heterogeneity and complexity, represents a force of aggregation that works at several different levels of matter structure (photons/atoms/molecules/cells/organisms) when abiotic structures come in close contact and interact with biological ones. Finally, life follows an “exponential” law of matter aggregation and compaction that resembles that of gravitation (a gravitation-like effect). Notably, living systems seem to be able not only to produce matter compression, but also able of time compression. A process in which the sequence events are speeded up and presented in a much shorter time than in the normal flow of time. Indeed, I have already hypothesised that the phylogenetic route of a species, a process that takes million years, could be retraced in a process of few weeks or months during embryogenesis (see Section 7.2). To summarize the evolutionary history of a species during ontogeny, it is likely that “epigenetic memories” may contain only the successful events that have led to current species; similarly, a book of physics mainly contains only the successful theories and compresses 350 years of studies in a text that can be learned in much less time, suggesting that time compression could be a common feature present at different levels of living systems. Based on the assumption of a time compression process occurring in living systems, more than twenty years ago I had hypothesised the presence of relativistic effects affecting the flow of time in highly proliferating cells. My hypothesis was that a high speed of cell proliferation and therefore of atom movement in biological structures could slow down the flow of time (time dilatation) as in the famous twin paradox associated with Einstein’s theory of relativity. To investigate this possibility, I did preliminary experiments using radioactive decay as an internal clock. K562 leukaemic cells were loaded with radioactive ^32^P-orthophosphate and radioactivity of living and killed (with freeze-thaw or detergent treatment) cells was detected at different (0,2,4,6) days of culture. Unexpectedly, radioactive emission of living cells was reproducibly lower than that of dead cells (Zamai unpublished observations), suggesting that proliferating cells might be able to produce a “relativistic” acceleration of the time flow (as it seemingly occurs during embryogenesis) or, alternatively, that living organisms were able to absorb more energetic electrons (products of ^32^P beta decay) than abiotic matter (as occurs with energetic UV photons), finally reducing the radioactive emission detected by beta counter. In line with this last hypothesis was the observation that when the living cells spontaneously die (after 4–6 days of culture), their radioactive emission reached levels comparable to that of the cells killed at the onset of the experiment; a phenomenon that, on the other hand, produced an apparent slowdown of the time flow (a black hole-like effect). Obviously, this is only a preliminary observation that needs careful verification before drawing conclusions. Nevertheless, the presence of life, a unique “physical” force of aggregation, makes the Earth a “privileged” and unique viewpoint of the Universe. Therefore, we have to consider that the “singularity” of our position of observation might influence the astronomical measurements of the Universe. Indeed, life is a singularity, a second big bang that, however, aggregates instead of dispersing matter, therefore working in a black-hole-like fashion. Black hole is considered a gravitational singularity and the singularity of living systems acts as a gravitational-like force, raising the incredible possibility that black hole-like signals might hide the presence of living systems and/or that the Earth might be the birthplace of a black hole-like system. A condition that could generate an increase in curvature of the space-time and, resembling the apparent motion of the sun, an Earth’s apparent motion away from the rest of Universe (or vice versa), as suggested by Einstein’s theory of relativity.

In this regard, I am working on a project that is trying to shed light on some weird physical aspects of matter compaction proposing a new model of Universe based on two immiscible phases, matter and space (as water and oil). Based on this model, during the matter diffusion into the space after the Big Bang, the Universe had acquired potential energy which is now exemplified by the gravitation force that locally induces matter compaction and formation of celestial bodies (as drops of oil dispersed into water tend to progressively aggregate). In this view, life, being a physical force of compaction, is perfectly in tune with the hypothesised process.

Finally, I would like to emphasize that my multidisciplinary approach to the study of evolutionary processes is not a philosophical one but rather supported by a wealth of analytical data. Indeed, the mechanisms unveiled in the present work are based on biological and chemical-physical mechanisms and not on any teleological “explanation”. Importantly, the examples I have discussed are viewed as the result of chance and natural selection and not the consequence of something that was planned. However, I would be grateful for any constructive criticism or corrections that might improve my personal and limited scientific view. Notably, the mechanisms unveiled raise new issues and offer novel perspectives which could have repercussions in a wide range of scientific fields. I am confident that the rational approach based on logical precepts adopted in the present paper will ultimately be appreciated by the scientific community. This work is a sort of message in a bottle that, sooner or later, I hope will go “viral” and inspire other scientists to make improvements in their respective scientific fields and to explore new areas of research. Personally, I already have in my mind new strategies to prevent and counter chronic diseases and a new model of the Universe based on two immiscible phases that I would like to describe in future works.

If we can improve our understanding of where we come from and how we came to be, we can get a more complete picture of the present and perhaps even foresee the future. Otherwise we will only have an instantaneous and incomplete picture of the present.

## Abbreviations

Activation-induced deaminase	AID
Adenosine	A
Adenosine deaminase enzymes acting on RNA	ADARs
Androgen receptor	AR
Androgen response elements	AREs
Apolipoprotein B mRNA editing enzyme, catalytic polypeptide-like	APOBEC
Clustered, regularly interspaced, short, palindromic repeats	CRISPR
Cytosine	C
CRISPR-associated	Cas
CRISPR-associated complex for antiviral defence	Cascade
CRISPR RNAs	crRNAs
Dihydrotestosterone	DHT
Ecological evolutionary developmental biology	Eco-Evo-Devo
Endogenous retroviruses	ERVs
Gene copy number variations	CNV
Guanosine	G
Haemoglobin	Hb
Heavy-chain class-switch recombination	CSR
Immunoglobulin	Ig
Inosine	I
Interferons	IFNs
Long interspersed nuclear elements	LINEs
Long-terminal repeats	LTR
Open reading frame 1 and 2 proteins	ORF1p and ORF2p
Protein	P
Short interspersed nuclear elements	SINEs
Single nucleotide polymorphisms	SNP
Single-stranded DNA	ssDNA
Somatic hypermutation	SHM
Stress induced mutagenesis	SIM
Switch	S
Thymidine	T
